# Motivations, barriers and exercise preferences among female undergraduates: A need assessment analysis

**DOI:** 10.1371/journal.pone.0264158

**Published:** 2022-02-28

**Authors:** Mohd Sham Othman, Arimi Fitri Mat Ludin, Lew Leong Chen, Hanisah Hossain, Ida Irwani Abdul Halim, Mohd Jamil Sameeha, Ahmad Rashidi Mohamed Tahir

**Affiliations:** 1 Center for Toxicology & Health Risk Studies (CORE), Faculty of Health Sciences, Universiti Kebangsaan Malaysia, Kuala Lumpur, Malaysia; 2 Biomedical Science Programme, Faculty of Health Sciences, Universiti Kebangsaan Malaysia, Kuala Lumpur, Malaysia; 3 Center for Healthy Ageing and Wellness, Faculty of Health Sciences, Universiti Kebangsaan Malaysia, Kuala Lumpur, Malaysia; 4 Nutritional Sciences Programme, Faculty of Health Sciences, Universiti Kebangsaan Malaysia, Kuala Lumpur, Malaysia; 5 Centre for Community Health Studies (ReaCH), Faculty of Health Sciences, Universiti Kebangsaan Malaysia, Kuala Lumpur, Malaysia; 6 Faculty of Pharmacy, University of Cyberjaya, Cyberjaya, Selangor, Malaysia; Universiti Malaysia Terengganu, MALAYSIA

## Abstract

**Introduction:**

The decreasing level of physical activity among female undergraduates is worrying as it is associated with the increased risk of non-communicable diseases. Thus, this study aimed to identify the motivations, barriers and preferences towards exercise among female undergraduates in Malaysia.

**Methods:**

A non-probability purposive sampling was used for the recruitment process. The inclusion criteria of the participants were registered female undergraduates and aged between 18–30 years old. A semi-structured in-depth interview was used to collect topic-related information from the participants and signed consents were obtained prior to the interview. The interview questions were on respondents’ understanding of exercise, motivation and barriers to exercise, and exercise preferences. The recruitment process was conducted until the data was saturated. All interviews were audio recorded and manually transcribed verbatim. NVivo 11 was used to conduct the inductive analysis of the data to develop themes for motivation and barriers to exercise. For exercise preferences, four predetermined themes were used.

**Findings:**

A total of 26 respondents participated in this study. Eight themes were found for motivation to exercise, with the most common themes being maintaining or improving appearance, health benefits and togetherness. For barriers of exercising, five themes were found, and the most common ones were disliking exercise and no motivation. For exercise preferences, most respondents preferred a structured exercise program with flexibility in terms of when and where the exercise could be conducted. Light or moderate intensity exercise for 10–30 minutes with a frequency of 1–2 times a week was desired the most among the respondents.

**Conclusion:**

In conclusion, personal and environmental factors play important roles in motivating or hampering female undergraduates to exercise, and a structured program was the preferred mode of exercise of these respondents. A new exercise module was designed based on this needs assessment with a 70% acceptance rate among the participants. These findings can help the future development of more exercise modules tailored to female university students.

## Introduction

Physical inactivity has been identified as one of the main factors of death globally [1]. Physical inactivity is defined as doing very little or no physical activity at work, at home, for transport or during discretionary time [2]. A study conducted by Hallal et al. [3] showed that 31.1% of the world population and 17% of the South East Asia (SEA) population were physically inactive. According to the World Health Organization (WHO) database in 2016, Malaysia ranked second for insufficient physical activity among adults in ASEAN countries [4]. Based on findings from the Malaysian Adult Nutrition Survey, there is a 25.1% prevalence of overall physical inactivity among adults in Malaysia [5]. The survey highlighted that Malaysians spent a lot of their time for sedentary activities such as sitting and lying down [6]. Furthermore, studies also showed that females were less active compared to males [3, 7, 8]. Based on a report by Hallal PC et al., the global prevalence of physical inactivity among females was higher (33.9%) compared to males (27.9%) [9]. This situation is similar in Malaysia where there is a significant difference between genders where males demonstrate higher levels of physical activity than females [9].

A previous study reported a decrease in physical activity participation during the transition to higher education [10], while a meta-analysis of 49 studies reported that physical activity level decreases when transitioning from adolescence to adulthood [11]. This was illustrated by Kwan et al. [12] who found that the highest decrease in physical activity levels occurred during university admission. This is further supported by several other similar studies on university students [13–15]. The data from the Malaysian National Health and Morbidity Survey reported that there is a 31.5% prevalence of physical inactivity among Malaysians between the ages of 15–24, which was higher compared to the adult age group younger than age 55 which had a 16.1–22.3% prevalence [16]. This has become one of the contributors of the high number of overweight or obesity cases among Malaysian adults (50.1%) [17]. Consequently, college and university students are more susceptible to weight gain during the college/university transition period, which is contributed by increased calorie intake and decreased physical activities [18, 19]. Among tertiary level students, female students were found to have less engagement in physical activities compared to male students [20–22], as the female students often had lower motivation compared to male students [23]. This could potentially explain their failure in achieving the target of 10,000 steps daily, approximately similar to 150 minutes/week of moderate-intensity physical activity as recommended in several studies [24–27].

A lot of effort has been carried out to combat obesity and its associated risks in Malaysia. One of them is the large allocation of the healthcare budget (~USD 1.7 billion, 13.3% of 2017 healthcare cost). However, there is still a growing prevalence of overweight and obesity among Malaysian adults, from 47.7% to 50.1% over the years of 2015 to 2019 [17]. Hence, it is important to intervene to improve and develop effective exercise habits for female undergraduates during this period of transitioning from adolescence to adulthood as it is critical in determining their future well-being [28]. Physical exercise is commonly known as a subset of physical activity, and achieving the exercise recommendation is correlated with achieving the physical activity level recommendation as well [29]. To increase the level of physical exercise among female university students, it is important to explore their motivations, barriers and exercise preferences. Studies showed that the motivation to exercise [23, 30–32], exercise barriers and exercise preferences [7] were significantly associated with physical activity levels. Therefore, it is imperative that a needs assessment is conducted before any attempt to tailor an exercise module for female undergraduates. The main objective of this study was to conduct a needs assessment to identify motivations, barriers, and preferences towards exercise among local female undergraduates. This is the first study to only focus on Malaysian female respondents. Furthermore, all respondents were health sciences students who had basic health sciences knowledge. Therefore, the focus group in this study was female health science students only.

## Methods

### Sampling and recruitment

This qualitative study was conducted to explore motivations, barriers, and preferences towards exercise among female undergraduates in Universiti Kebangsaan Malaysia, Malaysia. Based on the recommendation by Dworkin [33], a sample size of 25–30 is adequate to reach data saturation and redundancy in studies that utilize in-depth interview. A non-probability purposive sampling was employed to recruit 25–30 female students from Universiti Kebangsaan Malaysia. The inclusion criteria were a) female; b) officially registered students during the study period, and c) aged between 18–30 years old. Physically disabled and students suffering from mental health problems were excluded from the study as the challenges and barriers for them to perform exercise might not be similar to our study population. The recruitment and interview were conducted until the data reached saturation.

### Ethics statement

Ethical approval was obtained from the Universiti Kebangsaan Malaysia Human Research Ethic Committee (UKM PPI/111/8/JEP-2017-656). Respondents were briefed about the purpose and the procedures of the study. Informed written consent was obtained from each respondent prior to the interview.

### Interviews

The interview protocol used in this study was adapted from Lees et al. [34] and was pre-tested on five female students. Amendments to the questionnaire script were done prior to the subsequent interviews. The interview was conducted by researchers, Hanisah Hossain (HH) and Ida Irwani Abd Halim (IIAH) in either English or Bahasa Malaysia according to each respondent’s preference. Respondents were asked questions regarding their exercise habits. Respondents who exercised regularly were asked about their reasons for exercising regularly, motivations to keep exercising regularly and barriers hindering them from exercising. Respondents who did not exercise regularly were asked about their barriers or reasons for not exercising and motivations for them to start exercising. Both groups of respondents were also asked about their understanding on exercise and their exercise preferences based on the frequency, intensity, duration and types of exercise. To assist the participants, slow jog, casual walk, and Tai Chi were provided as examples of low intensity exercise, whilst for moderate intensity the examples given were brisk walk, dancing, biking, and the examples of high intensity exercise were group exercise circuit training, running, and competitive ball sports. The complete interview question guide is attached in S1 File. Each interview session lasted for about an average of 30 minutes and was conducted face-to-face at the residential college between January to May 2017.

### Data handling and analysis

Each interview session was recorded using a smartphone recording application and was transcribed verbatim. Both inductive and deductive thematic analysis were employed in this study. For motivation and barriers towards exercise, a qualitative data analysis program, QSR International NVivo version 11 was utilized to analyze the themes [35, 36]. Data triangulation method was used to analyze the data [37, 38]. The first step of the data analysis was reading and entering the transcribed verbatim into NVivo 11 where the data was coded by two members of the research team (HH and IIAH). The codes were then consolidated according to the research question. The second step was reviewing the identified codes by two other researchers (AFML and MSO) to further consolidate the number of codes into categories and to identify relevant main themes that answered the research questions. The third step was assembling the themes to form more general concepts. Data validity was confirmed when the themes were repeated in the data analysis. A final meeting was held with the rest of the researchers to finalize all the codes, categories and themes. To ensure that the data analysis remained neutral and was not influenced by themes from other studies, literature research was conducted at the end of the study. For exercise preferences, responses from the respondents were classified into four predetermined themes which were frequency, intensity, time, and type of exercise preferred.

## Results

### Respondents characteristics

30 female respondents were recruited for this study, but information had reached saturation at the 26th participant. The 26 respondents were between 21 to 27 years old (mean age 23.00±1.17 years old). The respondents consisted of Malays (61.5%), Chinese (15.4%) Indians (7.7%) and other ethnicities (15.4%). The study location was the health campus at University Kebangsaan Malaysia; hence all the respondents were from healthcare related courses: nutrition, environmental and health sciences, biomedical sciences, optometry and dentistry programs. The complete sociodemographic characteristics of the respondents are shown in Table 1.

**Table 1:** Sociodemographic information of respondents.

Subject Code	Age	Race	Religion	Program/Year
R1	23	Dusun	Christian	Nutrition/4
R2	23	Chinese	Buddhist	Biomedical Sciences/4
R3	23	Indian	Hindu	Biomedical Sciences/4
R4	23	Malay	Muslim	Biomedical Sciences/4
R5	23	Malay	Muslim	Nutrition/4
R6	26	Malay	Muslim	Biomedical Sciences/4
R7	27	Malay	Muslim	Biomedical Sciences/4
R8	23	Malay	Muslim	Biomedical Sciences/4
R9	21	Malay	Muslim	Dentistry/2
R10	23	Malay	Muslim	Biomedical Sciences/4
R11	23	Malay	Muslim	Dentistry/4
R12	23	Chinese	Buddhist	Biomedical Sciences/4
R13	23	Malay	Muslim	Optometry/4
R14	23	Chinese	Buddhist	Biomedical Sciences/4
R15	23	Indian	Christian	Dentistry/2
R16	23	Chinese	Buddhist	Biomedical Sciences/4
R17	22	Bajau	Muslim	Biomedical Sciences/4
R18	23	Malay	Muslim	Dentistry/3
R19	22	Malay	Muslim	Environmental Health Sciences /4
R20	23	Malay	Muslim	Biomedical Sciences/4
R21	22	Malay	Muslim	Environmental Health Sciences /4
R22	23	Malay	Muslim	Biomedical Sciences/4
R23	23	Malay	Muslim	Optometry/4
R24	23	Malay	Muslim	Biomedical Sciences/4
R25	22	Bugis	Muslim	Nutrition/4
R26	23	Kadazan	Christian	Nutrition/4

### Motivation to exercise

Multiple factors motivated respondents to start and continue exercising in their daily life. In this study, eight themes emerged as motivation towards exercise, as shown in Table 2. They were: Maintenance or improvement of physical appearance, health benefits, togetherness, coach or trainer, discipline or commitment, facilities, enjoyment, and stress release.

**Table 2:** Motivation to perform exercise among respondents.

Theme	Subtheme
**Maintain/ Improve Appearance**	Weight gain
	Lose weight
	Muscle tone
**Health Benefits**	Improve fitness
	Maintain/ Improve Physical Health
	Informed of Health Benefits
**Togetherness**	Be with friends
	Group Activity
**Coach/ Trainer**	
**Discipline/ Commitment**	
**Facilities**	Convenient place to exercise
	Appropriate exercise equipment
**Enjoyment**	
**Stress Release**	

#### Theme 1: Maintain/improve physical appearance

Over half of the respondents stated that their main motivator to exercise was to maintain or improve their physical appearance (n = 14). When probed further, respondents intended to increase weight (n = 5), reduce body weight (n = 6) and body shape/ muscle tone to increase attractiveness (n = 3). One of the respondents, R25’s (Bugis, Muslim, 22) statement is as follows:

“*Actually*, *the main reason that I want to exercise is to reduce my body weight because of my fat*. *I have a high fat concentration*, *so I want to shape some muscles*. *Because every time I check*, *it’s always higher than it should be (fat)*. *So that’s the real reason that I want to lose body weight…*”.

#### Theme 2: Health benefits

Health benefits were mentioned by 13 respondents. The respondents desired to improve fitness levels (n = 6), maintain or improve physical health (n = 5) and to know the health benefits of the exercise prior to exercising (n = 2). Some respondents were keen to improve physical health that it outweighed the hindrance to exercise, including pain. An example of such response came from respondent, R6 (Malay, Muslim, 26): “*When there is a change in my lifestyle like when I’m not even tired after going up and down the stairs*. *My body feels more fit and I will definitely feel more addicted to exercise*. *To me*, *I’m hooked*. *For example*, *with tae-kwando*, *my body only hurts for 2 weeks and then we start with movement week*, *it’s great*. *Just great*”. Other respondents talked about being active previously but were currently living a sedentary lifestyle. Hence, they aspired to be healthy and active again. For example, R5 (Malay, Muslim, 23) stated that, “*I want to be fit because I used to play handball a lot and back then*, *my body felt so great*. *I don’t know how to explain it but it’s great*. *When I wake up I feel great but now that I don’t play as much*, *I get tired easily*. *So*, *to be fit again would be great*”. Some respondents wanted to be fitter than their current state and expressed that they would exercise more often if they had someone to teach them. This was mentioned by respondent, R6 (Malay, Muslim, 26), “*Maybe if I could be friends with someone who knew about exercise or somebody else who knows how to exercise*, *I would*”.

Being students, academic performance had become their priority and it had outweighed the perceived health benefits from exercise. Some respondents reported to have busy schedules, hence were selective on how they spent their time. Others wanted to know the specific health benefits of the exercise prior to exercising. For example, respondent R2 (Chinese, Buddhist, 23) said, “*If I know how the exercise activity or the exercise module can benefit me*, *like what I can get in return*, *only then am I motivated to do it*”. Some respondents wanted dietary advice and energy expenditure estimation in the exercise module that would be developed in the future.

#### Theme 3: Togetherness

The third theme, togetherness (n = 10) was further divided into spending time with friends (n = 6) and joining a group activity (n = 4). Respondents’ motivation was influenced by the presence of their friends. For example, respondent R19 (Malay, Muslim, 22) mentioned that “*Um*, *for motivation*, *if someone invites me to exercise then I can follow them but if no one invited me to exercise*, *I will definitely not exercise by myself*”. Another example is R10 (Malay, Muslim, 23) who said, “*It’s like a support system*, *I think*, *when we exercise with friends*, *we have a gang*”. Other respondents preferred joining a group activity. One respondent, R22 (Malay, Muslim, 23) remarked, “*Because there are many people in a group*. *The more people there are*, *the more motivated I feel*. *When we see other people (become) motivated*, *we also feel like*, *oh*, *we want to do that too but when we are alone*, *we feel like lazy*, *do tomorrow and tomorrow*. *We procrastinate it until we eventually end up not doing it*”.

#### Theme 4: Coach/trainer

The availability of a coach or a trainer was mentioned by the respondents as a motivating factor for them to exercise (n = 4). One respondent, R13 (Malay, Muslim, 23) said, “*It would be easier if there was someone to teach me to exercise and what to do*. *If I’m alone*, *it is much harder to learn how to exercise*”.

#### Theme 5: Discipline or commitment

The best example for the theme discipline or commitment (n = 4) as a motivator to exercise was this response: “*For me*, *exercise is like a discipline*, *we have discipline to keep moving us*, *to do the exercise regularly*. *So*, *and the discipline can be applied in all the other stuff*, *in our daily life*, *so I feel like this is my responsibility to do the exercise*”. Therefore, exercise is perceived as an activity to develop positive character traits.

#### Theme 6: Facilities

The sixth theme for motivation to exercise is facilities (n = 4). Facilities include convenient place to exercise (n = 3) and appropriate exercise equipment (n = 1). Respondents’ motivation to exercise was dependent on the quality and availability of facilities in the residential college. For example, R10 (Malay, Muslim, 23) mentioned, “*If the place to exercise is satisfactory then I am interested to exercise there*. *That’s what can add motivation for me to exercise*”. As for appropriate exercise equipment, one of the respondents, R21 (Malay, Muslim, 22) said, “*If it was like before this*, *when I had mood to exercise*, *maybe I would buy a skipping rope*, *put it in my room so that I can use it every day*. *That’s all that I can think of that can increase my mood to exercise*. *We can buy exercising equipment to help us exercise*”.

#### Theme 7: Enjoyment

Enjoyment towards exercise was also a motivating factor for some of the respondents (n = 4). One respondent, R5 (Malay, Muslim, 23) said, *“Sometimes exercise makes me happy*. *Feel happy especially when we enjoy the exercise”*

#### Theme 8: Stress release

The final theme associated with motivation to exercise is its stress releasing capability (n = 1). Only one respondent mentioned that exercise can help reduce stress from studying in the room for a prolonged time. The respondent R15 (Indian, Christian, 23) said, “*Because exercise release stress*, *some more if you stuck in your room for so long you will feel stress facing the four walls*”.

### Barriers to exercise

As shown in Table 3, five themes were identified as barriers to exercise among the respondents. The themes were exercise animosity, no motivation to exercise, time constraint, discomfort or pain during or after exercising, and lack of facilities.

**Table 3:** Barriers to perform exercise among respondents.

Theme	Subtheme
**Dislike exercise**	Dislike exercise in general
	Dislike exercising alone
	Dislike being seen exercising
**No motivation**	No motivation
	No mood
**Time Constraints**	
**Discomfort/ Pain During or After Exercise**	Fatigue
	Pain after exercise
**Lack of Facilities**	

#### Theme 1: Dislike exercise

Most of the respondents did not exercise because they disliked exercise or physical activity. This theme is divided into three categories which are general dislike towards exercise (n = 4), dislike being seen exercising (n = 5) and dislike exercising alone (n = 3). For general dislike towards exercise, respondents stated they did not like exercise and that exercise did not make them happy. For example, R21 (Malay, Muslim, 22), “*Because when you exercise*, *you sweat*, *then you have to change your clothes and shower…its extra effort*, *I’m lazy and I don’t like it*”.

As for dislike being seen exercising, all the respondents who reported this as a barrier were Muslim respondents. One of the respondents said that she did not have self-confidence. Another respondent R5 (Malay, Muslim, 23) said, “*When exercising outdoors*, *I feel a bit ashamed when exercising with people who are already fit and I*, *on the other hand*, *am not*”. One accurate example for this theme is described by respondent R22 (Malay, Muslim, 23): “*I actually like exercise*, *but I don’t like it when people watch me exercise*. *I seriously don’t like it*, *not at all*. *Sweating is fine and all it’s just that I don’t like it when people watch me exercise*”. For the category dislike exercising alone, one of the respondents, R10 (Malay, Muslim, 23) said, “*I have no friend to go exercising with*. *No gang and then if I exercise in my room*, *I also have no company so it’s not fun*”.

#### Theme 2: Lack of motivation

Another major barrier to exercise is the lack of motivation (n = 11). Respondents were not motivated to join sporting events, exercise by themselves or with friends either indoors or outdoors (n = 10). One respondent said that she did not have the mood to exercise. R21 (Malay, Muslim, 22) said, “*I’m lazy*, *I’d rather waste time watching stories on my laptop than exercise*”. Another respondent, R10 (Malay, Muslim, 23) said, “*Because if I exercise in the morning*, *um…I’m lazy to wake up in the morning*”. One respondent, R11 (Malay, Muslim, 23) even said, “*These days I rather sit down rather than walk and do exercise*”.

#### Theme 3: Time constraints

The next theme is time constraint (n = 10). Majority of the respondents depended on the bus provided by the university on weekdays, and public transport such as e-hailing services and city transit buses to commute. Based on the observation and experience of the researchers, students would usually take the bus departing to the faculty early in the morning and return with the campus bus late in the evening. Even though the distance between the faculty and residential college is not far, heavy traffic during peak hours would increase the travel time of the respondents. Apart from that, respondents also tend to spend more time at the college’s cafeteria. This is because there is only one cafeteria operator and the waiting time for food is quite long. In addition, students are also expected to be involved in college activities and most of them are non-exercise related. Many respondents needed to have good time management for academic responsibilities, co-curricular activities and exercising. In many cases, exercising was often the lowest priority. One of the respondents, R14 (Chinese, Buddhist, 23) said, “*If the academic work is too heavy or activity or co-curricular is too many then I will skip*, *I may skip the exercise*”.

#### Theme 4: Discomfort/pain during or after exercise

Most of the respondents reported experiencing discomfort or pain during or after exercise (n = 10). This was another deterrent to exercise. Fatigue (n = 7) and body aches after exercise (n = 3) are coded into this category. An example for fatigue was mentioned by respondent R19 (Malay, Muslim, 22), “*Sometimes when we exercise in the afternoon*, *by the time we get back to our room we are tired*. *So*, *at night cannot do that much work*. *Sometimes I even fall asleep*”. Respondents said that exhaustion from classes and the travel between faculty and residential college in the evening were the main obstacles that prevented them from exercising. As for pain after exercising, respondent R6 (Malay, Muslim, 26) said that, “*The thing about exercise that I don’t like is that after exercise*, *especially when the body is just beginning to adapt to exercising*, *I’ll get body aches and pain after*”.

#### Theme 5: Lack of facilities

The final theme is lack of facilities (n = 1), and respondent R10 (Malay, Muslim, 23) clarified this, “*I used to like going to the gymnasium but now because the equipment and the environment in the gym isn’t good*, *I don’t go anymore*”. Based on the experience and knowledge of our researchers who are also UKM students, the residential colleges in UKM provided sports equipment mainly for sports programs sanctioned by college management, and personal utilization of equipment required written permission. While the college gymnasium is accessible to every resident, its availability to female undergraduate residents was restricted. Female residents are only allowed to use the gym on selected days of the week. To make matter worse, many of the gym’s equipment were defective and the number of equipment was insufficient.

### Exercise preferences

Exercise preferences refer to the individually preferred choices and situations that encouraged respondents to exercise. The feedback from respondents was classified according to the FITT principle, a framework used in exercise prescription as suggested by the American College of Sports Medicine [39]. The FITT principle includes frequency, intensity, time and type of exercise preferred. Frequency refers to how often exercise is normally performed in a week. Intensity indicates how difficult it is to perform the exercise. Time refers to the duration of the exercise performed, and type represents the mode of exercise.

The exercise preferences by the respondents are shown in Table 4.

**Table 4:** Exercise preferences among respondents.

Theme	Subtheme
**Frequency**	1–2 times/week
	3 times/week
	4–5 times/week
**Intensity**	Low intensity
	Moderate intensity
	High intensity
**Time**	Less than 10 minutes
	10–30 minutes/session
	More than 30 minutes
**Type**	Structured exercise program
	Flexible in time & place
	Group Activity
	Individual

In terms of frequency, majority of the respondents (n = 21) chose to exercise once or twice a week while only a minority would exercise three times a week (n = 5). None of the respondents were keen to exercise more than three times a week. Interestingly, one of the respondents, R22 (Malay, Muslim, 23), chose to be flexible in determining the exercise frequency by admitting, “*Um*, *to me that isn’t too frequent*. *It depends on how busy I am as well*. *If I’m not busy*, *exercising every 2 days is fine*”.

In terms of intensity, more than half of the respondents preferred to perform exercise with moderate intensity (n = 15) while the rest chose to do light intensity exercise (n = 11). None of the respondents wanted to do high intensity exercise. For example, respondent R18 (Malay, Muslim, 23) mentioned, “*When it comes to going up and down the stairs*, *confirm I feel so lazy*. *If there was a lift*, *I would definitely take the lift*. *I can’t bear to do high intensity exercise*”.

As for exercise type, most respondents seemed to prefer individual exercise (n = 17) compared to group activity exercise (n = 9). Their favorite exercises were planking, jogging at the park, and *Zumba*. Some respondents desired exercises that could be done in a confined space like their own room (e.g. *Zumba* following YouTube^®^ videos) while others preferred to exercise outdoor. One of the respondents, R12 (Chinese, Buddhist, 23) said, “*If I had a friend who had similar interests with me*, *I would rather exercise in a group because we can give each other motivation because we are friends*”. Respondents requested the inclusion of interesting exercise activities should an exercise module be produced. For instance, one of the respondents said, “*The module has to be interesting*. *Do Zumba together with music for free*”.

In short, respondents wanted a simple, easy structured exercise module that was flexible in terms of time and place. Respondent R22 (Malay, Muslim, 23) elaborated that, “*Maybe aside daily supervision to check if a person exercises*, *maybe you can make a schedule*. *Today*, *what do you have to do*, *how many sit-ups do you have to do*. *The thing has to be organized*. *How many times do you have to do something*, *how many minutes do you have to do it*, *how do you do it*, *like that*. *Like*, *I actually want to exercise but I don’t know how*, *like for this*, *should I do this thing first*, *or that thing first*. *Should I do sit ups first*, *or this other thing first*. *I don’t know the basics of exercise*. *I do watch videos on YouTube*^®^ *and others but sometimes I do them and other times I don’t*. *If we can produce something that’s like organized and okay*, *God willing*, *I can follow it*”.

For the preferred duration of a session of exercising, more than half of the respondents chose to exercise within 10–30 minutes per session (n = 14). Some of the respondents (n = 8) wanted to exercise less than 10 minutes at a time while the rest (n = 4) agreed to exercise more than 30 minutes per exercise session. One of the respondents, R1 (Dusun, Christian, 23) said, “*When it comes to doing exercise*, *it depends on the timing as well and how it suits with our schedule*. *In terms of time*, *maybe I can allocate about 30 minutes to an hour*, *it depends*”.

## Discussion

Majority of the respondents preferred to exercise individually, with low to moderate intensity exercises that last 10–30 minutes per session, with frequencies of 1–2 times a week. However, these preferences do not meet the WHO global recommendation for physical activity, nor the Malaysian Ministry of Health (MOH) exercise guidelines as stated in Malaysian Dietary Guidelines [40]. MOH recommends that Malaysians should conduct moderate intensity exercise for at least 5 days a week and a minimum of 30 minutes for each session. This exercise recommendation is also in line with the recommendation by WHO, Malaysian Adult Nutrition Survey [41] and a study by Poh et al. [5]. Although our respondents’ exercise preferences did not meet the minimum recommendation, they were engaged in some level of physical activity. Studies have shown that any duration of physical activity is better than none, and there is a dose-response relationship between physical activities and associated risk reduction in metabolic syndrome, premature mortalities and mental health problems [42–45].

For exercise intensity, the selection of lower intensity exercise compared to high intensity exercise might be caused by the less pleasurable or negative feelings associated with high intensity exercises [46, 47]. Pain and discomfort were mentioned by majority of the respondents as one of the barriers that prevented them from exercising. The target group preference towards exercise intensity is an important aspect to consider when developing future exercise modules as it can affect individual’s interest to continually engage and adhere to the exercise program. Intensity of exercise should be gradually increased to achieve better adherence to the exercise routine as recommended by American College of Sports Medicine [48]. A majority of the respondents also stated the need for an easy and flexible structured exercise program which can be done individually.

Our study identified several themes for motivation to exercise which were maintenance or improvement of physical appearance, health benefits, togetherness, coach or trainer, discipline or commitment, facilities, enjoyment and stress release. Our respondents’ main motivation to exercise was to maintain or improve physical appearance. This was in agreement to other studies which reported that the desire to improve appearance and body image dissatisfaction were associated with practicing physical activities [49–52]. A study on college students related that appearance was one of the extrinsic factors that positively influenced the students to exercise [32]. This finding was also supported by Baker [53] who stated that the motivation to exercise was dependent on a woman’s attitude towards body weight and dieting, and also their perception of their own body image. Negative perception towards obese individuals is common among the community nowadays [6]. Obese people often experience discrimination and poor treatment from the society in various aspects of their daily life such as in education, at work and in romantic relationships [6]. Research has shown that people who experience weight stigma are likely to be impacted with significant negative psychology like depression, dissatisfaction with their body image and low self-esteem [54–56]. While weight stigma can decrease the motivation to exercise [57], maintaining and improving physical appearance were the main motivators amongst the respondents in this study. This was probably to avoid experiencing weight stigma or to disassociate themselves from past experiences with weight stigma. Exercising with an intent or objective related to self-appearance is associated with negative perceptions of their own body image for female adults and youth [58].

Health benefits is the second most chosen motivator by the respondents for exercising. The desire to maintain or improve their health fitness influenced the respondents to conduct physical activities. Previous studies have reported that good fitness, weight management and perceived health benefits were major motivators for individuals to exercise [31, 52, 59–62]. A study carried out among adults in the Philippines reported that their main motivation for exercising was to maintain their overall fitness and for weight management [50]. In conclusion, more education and awareness should be imparted to students in universities or colleges regarding the advantages of exercise to help increase participation in physical activities.

Another motivator mentioned by some of the respondents was togetherness. Respondents felt more motivated when they exercised with their friends. This was similar to another study which showed that socializing with peers and receiving support or influence from family members could be a motivator for exercising [63]. In addition to that, social support and enjoyment were also identified as motivators for physical exercise in several studies [59, 64, 65]. Young adults tend to be more motivated by togetherness and when affiliated with sports team mates compared to older adults [30]. A recent research showed that face-to-face structured weight reduction intervention with group exercise sessions had a larger effect on weight loss compared to online intervention [66].

Based on a research done on university students to discover their motivation towards exercise participation, the top motivations for female students’ participation in exercise were stress management, revitalization, health benefits, weight management and appearance. These were comparable to the findings of our study, where the overlapping motivations were health benefits and weight management [67]. This was in agreement with two other studies which reported that female university and college students were more driven by extrinsic motives towards exercise, such as weight management and appearance [68, 69].

According to Molanorouzi [30], there are eight motivating factors towards exercise among younger adults. The factors are mastery, enjoyment, psychological condition, physical condition, appearance, other’s expectation, affiliation and competition or ego. However, Rogers et al. [70] only listed seven meaningful motivations in exercise which were competition/ego, extrinsic rewards, social health, physical health, psychological health, mastery of a sport, and enjoyment. While both studies had different numbers of motivating factors, many of the factors overlapped between the two studies. In many cases, motivation to exercise is not determined solely by one factor. Often, the motivation to exercise is due to a multitude of linked factors. More research is needed to find the association between these factors and physical activity and to what extent these factors may be able to predict physical activity.

The main barrier to exercise in this study was the feeling of apprehension to exercise which included general exercise animosity, dislike being seen exercising, and dislike exercising alone. Sechrist et al. [71] reported similar barriers to exercise among male and female adults aged 18–88 years old. The study reported that common reasons included perceiving exercise as a highly embarrassing activity, the awkwardness of wearing sportswear, the inconvenience to travel to an exercise facility and time constraint. The same study reported exercise as an activity that required hard work and was tiresome. Some of the respondents in our study also disliked being seen exercising. Self-consciousness or social discomfort about participation in exercise programs is a common barrier among women [72, 73]. This highlights the need for a different set of exercise module to be developed which can either be performed individually in private areas, or with friends to meet the preference of respondents.

Findings from this study also showed that time constraint was a major barrier for the respondents to conduct physical activities. University Kebangsaan Malaysia students tend to spend plenty of time travelling between campus to the residential college and to the only canteen operator in the college. They were also involved in a variety of non-exercise related activities in college and had academic responsibilities, hence, lowering the priority for exercising. Van Dyck et al. [74] reported that a low self-efficacy and time-related barriers decreased students’ participation rate in leisure sports. Similarly, time-related barriers have been reported in numerous studies as a hindrance towards exercising [72, 75, 76]. Interventions focusing on time management and improving self-efficacy can be beneficial to encourage the students to be more involved in physical activities. Recommendation to incorporate short bouts of exercise between living activities can be advocated to the students to increase their physical exercise.

Another major barrier to exercise in this study was lack of motivation. Laziness and lack of interest towards exercise have been reported as some of the major barriers that hindered students [77] and other age groups [75, 76, 78] from participating in physical activities. External motivators for individuals to exercise can also be important. The motivators can be in various forms, such as convenience and ease to access facilities, competitions and availability of trainers. The outcome from each physical activity will also contribute towards the motivation to improve physical level. In developing future modules, setting up goals can also motivate students to take up and adhere to the exercise modules. Interventions with set goals have been known to be effective in improving physical activity behaviors [79, 80].

Our findings in exercise barriers of female students were similar to another study which focused on female health care workers. The top two barriers were that they disliked the exercise apparel and they were embarrassed to exercise [81]. Another research conducted on Malaysian university students highlighted that time constraint is one of the major barriers that hindered students from exercising. They also discovered that although the students were interested in exercising, they lacked the required motivation [82].

It is important to fully understand the motivators, barriers and exercise preferences of female undergraduates towards exercise. There is a need for academic institutions to encourage exercise as an integral part of university or college life. Once motivators, barriers and exercise preferences are clearly identified, programs can be designed to maximize motivation factors and minimize barriers to physical activity among students. More importantly, exercise programs can be catered to the needs and wants of these female young adults. The information gathered from this study can help practitioners design effective exercise modules for female undergraduate students in the future.

## Limitation and strengths

This study had certain identified biases. The first bias was the respondents’ exposure to the benefits of exercise. All respondents were enrolled in health-related degree programmes. They may have higher perceived benefits of exercise compared to non-health related students. Secondly, the living condition of the respondents may have affected the outcome of the research. The respondents’ campus and residential college are located in the city center where traffic congestion often lengthens the commuting time. This may be different from students from campuses outside the city center. Thirdly, although the data reached saturation with 26 respondents, the result of this study cannot be generalized to all Malaysian universities. More studies are needed to ensure the generalizability of these findings.

In terms of strength, the one-to-one in-depth interview provided a safe and comfortable environment for the respondents to provide insightful feedback regarding the subject matter, as each respondent had the freedom to express their point of view without being influenced or intimidated by others. To our knowledge, this is the first study to explore the motivation, barriers and preferences toward exercise, specifically among female university students in Malaysia.

## Conclusion

This study unravels eight themes that were identified as motivating factors and five themes that were barriers to exercise. The motivation themes include maintaining or improving physical appearance, health benefits, togetherness, the availability of coach or trainer, discipline or commitment, facilities, enjoyment and stress release. On the other hand, exercise animosity, lack of motivation to exercise, time constraint, discomfort or pain during or after exercise, and lack of facilities constituted the barriers to exercise. Respondents showed the desire or motivation to exercise but often prioritized other aspects in their daily lives. The lack of drive to exercise can be attributed to the absence of companion (friend) to exercise, a suitable exercise program, convenient place to exercise, or lack of set goals to exercise. This study also found that respondents in this study prefer a structured exercise program that was flexible in terms of time and place of the exercise. Respondents also preferred low and moderate intensity exercise with the frequency of 1–2 times a week, and 10–30 minutes per session. An exercise module was developed based on the results of this needs assessment study and tested among female undergraduates with a 70% acceptance rate [83]. These findings can help the future development of exercise modules tailored to female university students to reduce physical inactivity among them.

## Supporting information

S1 File Interview question. (PDF)

S2 File Respondent interview transcripts. (PDF)
