# Supplementary material for: Motivations, barriers and exercise preferences among female undergraduates: A need assessment analysis
Source: PLoS One. 2022 Feb 28;17(2):e0264158. doi: 10.1371/journal.pone.0264158 (PMC8884489; doi:10.1371/journal.pone.0264158)
Supplement: S1 File — (PDF) [file pone.0264158.s001.pdf]

NEEDS ASSESSMENT INTERVIEW QUESTIONS (English Version)

Mandatory Question: Do you carry out regular exercise activities? If YES, use column A. If No, use column B.

A. Question for those who exercises	B. Questions for those who does not exercise
1. What do you understand about physical activity?<br>2. What do you understand about exercise?<br>3. When do you start exercising routinely and what is the main reason for doing so?<br>4. How many times do you exercise a week?<br>5. Is it enough for you?<br>6. What helps you to keep on exercising?<br>7. What will stop you from carrying out exercise?<br>8. In your opinion, what are the benefits of exercise for yourself?<br>9. If there is one exercise modules which requires you to exercise in one day intervals, are you willing to do so?<br>10. What can makes you maintain exercising in one day intervals?<br>11. Which type of exercise is your favourite?<br>12. What thing that you do not like about exercise?<br>13. We already know about your input on exercising. What are your favourite activities apart from exercising?	1. What do you understand about physical activity?<br>2. What do you understand about exercise?<br>3. What is your reason for not exercising?<br>4. What are the things that you do not like about exercise?<br>**5.** In your opinion, what are the benefits of exercise for yourself?<br>6. What is needed to be done for you to start exercising?<br>7. What can help you to maintain carrying out exercise?<br>8. Which type of exercise is your preference?<br>9. If we wanted to develop one structured exercise program. what will make you join the program and start exercising?<br>10. If there is one exercise modules which requires you to exercise in one day intervals, are you willing to do so?<br>11. What can makes you maintain exercising in one day intervals?

SOALAN TEMUDUGA PENILAIAN KEPERLUAN (Malay Version)

Soalan Wajib: Adakah anda ada melakukan aktiviti senaman secara rutin? Jika YA, guna kolum A, jika Tidak, gunakan kolum B.

C. Soalan untuk golongan melakukan senaman	D. Soalan untuk golongan tidak melakukan senaman
1. Apakah anda faham tentang aktiviti fizikal?<br>2. Apakah yang anda faham tentang senaman?<br>3. Bilakah anda memulakan senaman secara rutin dan apakah penyebab utama anda memulakannya?<br>4. Berapa kali anda melakukan senaman dalam satu minggu?<br>5. Adakah ianya sudah mencukupi untuk anda?<br>6. Apa yang membantu anda untuk kekal melakukan senaman?<br>7. Apakah yang akan menghalang anda untuk melakukan senaman?<br>8. Pada pendapat anda, apakah kebaikan senaman terhadap diri anda?<br>9. Jika satu modul senaman memerlukan anda untuk bersenam selang satu hari, adakah anda sanggup melakukannya?<br>10. Apa yang membuatkan anda kekal melakukan senaman selang sehari?<br>11. Apakah jenis senaman yang menjadi kesukaan anda?<br>12. Apakah perkara yang anda tidak suka tentang senaman?	1. Apakah anda faham tentang aktiviti fizikal?<br>2. Apakah yang anda faham tentang senaman?<br>3. Apakah sebab anda tidak melakukan senaman?<br>4. Apakah perkara yang anda tidak suka tentang senaman?<br>5. Pada pendapat anda, apakah kebaikan senaman pada diri anda?<br>6. Apa yang perlu dilakukan untuk anda memulakan senaman?<br>7. Apa yang membantu anda untuk kekal melakukan senaman?<br>8. Apakah jenis senaman yang menjadi kesukaan anda?<br>9. Jika kami ingin menghasilkan satu program senaman berstruktur, apa yang akan membuatkan anda untuk mengikuti program tersebut dan mula bersenam?<br>10. Jika satu modul senaman memerlukan anda untuk bersenam selang satu hari, adakah anda sanggup melakukannya?

13. Kita sudah mengetahui input anda tentang senaman. Apakah aktiviti kesukaan anda selain senaman?	11. Apakah yang akan membuatkan anda kekal untuk melakukan senaman selang sehari?
