# Supplementary material for: Motivations, barriers and exercise preferences among female undergraduates: A need assessment analysis
Source: PLoS One. 2022 Feb 28;17(2):e0264158. doi: 10.1371/journal.pone.0264158 (PMC8884489; doi:10.1371/journal.pone.0264158)
Supplement: S2 File — (PDF) [file pone.0264158.s002.pdf]

Supplementary File 2
Respondent Interview Transcripts (Translated from Malay language)

Transcript Respondent 1
Name: Anne
Age: 23 years
Faculty/Program/Year: FSK/Nutrition/4
Respondent Code: R1

I: Hello Anne. We are. I will be asking a few questions about your exercise activities> This session will be recorded. Can we start?

S: Sure

I: First question. Do you do activities regularly? Routinely? Like in a week, you exercise activities, it doesn't matter if it's just walking?

S: Not regularly. I do it according to mood

I: So it's not routinely done. Okay. What. What do you understand about physical activities?

S: Physical activities are activities or any activities that includes body movements

I: Body movements. Okay. The's physical activities. What do you understand about exercise?

S: Exercise. It's a planne physical activities

I: Planned?

S: We plan. Tes.

I: For example?

S: For example, jogging, cycling

I: We plan like?

S: meaning the thing we do. How do I say this. Things that we do according to our plan. It's not like daily movements that we do.

I: My next question, any reason why don't you exercise?

S: Laziness

Laziness. Like. Definition of laziness because of. Laziness because there's no place, or no time or no motivation?

S: Place and time are available. Maybe there's no motivation. Really lazy

I: Regarding exercise what don't you really like about exercise?

S: I do not dislike or, I'm just neutral about exercise

I: Just neutral. Lik eneutral. You don't like sweating. Don't like

S: No such thing

I: Or because it takes time

S: No. Just neutral

I: Just neutral. In your opinion, what… what do you think is the benefit of exercise?

S: Actually, I know about exercise. Like how it can help us get ideal body shape. And there's a lot of benefits. I know the benefits of exercise, it's just I don't have the motivation to do it. okay

I: All right. Let's say you want to start exercising. What could make us like, want us to exercise?

S: Aaaa

I: For example friends, or you have goals

S: In my opinion, if we want to start exercising, it should come from inside, we have our own goal, like own motivation. Because even if we have friends to exercise together, if we don't have motivation, we will not do it

I: If we start exercising to achieve our goal. How do me maintain the motivation to exercise? Like to keep on exercising?

S: Ooo. To maintain exercising is the reason why we exercise. We look at the benefit of.. For me, the benefits that we get from exercising. That's the way to maintain.

I: Like, example of benefits, like

S: What are the benefits. When we exercise, regularly exercise I will get some benefits from exercising, so when we see the benefits, that's why we want to continue

I: Thank you. Next, a question on.

S: Exercising..

I: What kind of exercise that you like

S: I Usually. If it was me. I usually go jogging or

I: Jogging

S: Jogging or cycling

I: Cycling. Anything else, like dancing

S: I seldom dance

I: Don't like?

S: Not really like it

I: Only jogging and cycling

S: Usually we can jog and cycle in group, enjoy in Lake Titiwangsa

I: Let's say we are making an exercise module. What would make you want to join the exercise program?

S: If you want a program, it depends on how interesting the program is

I: Interesting. Interesting in terms of games in the program or in the video?

S: Maybe I, how the program is conducted

I: Conducted like

S: I'm don't know actually, like what aa.. don't know how to describe interesting but..

I: Interesting to people

S: Maybe aa if the program has a coach. It requires someone who can attract people

I: If the module, requires you to do it every other day, would you do it?

S: Yes

I: willing to do it

S: This would depend on availability. Free time

I: Free time. When we have done it every other day, what would make you continue doing the exercise?

S: Like what I've mentioned before, the benefits that I would get from the exercise.

I: That's all the questions. Thank you

Additional questions

I: in the exercise module, which exercise intensity do you like, light, moderate of vigorous?

S: I like the combination of all three

I: In a week, how many days are you willing to exercise? And how long you can do it?

S: 3-5 times a week. When it comes to doing exercise, it depends on the timing as well and how it suits with our schedule. In terms of time, maybe I can allocate about 30 minutes to an hour.

I: If I were to develop the exercise module, how long, I mean how many months the module should cover?

S: About 24 weeks inclusive of resistance, flexibility and cardio

Transcript Respondent 2
Name: Leo
Age: 23 years
Faculty/Program/Year: FSK/Biomed/4
Respondent Code: R2

Hanisah: Hello, my name is Hanisah binti Hossain. You are here for a quick interview. I want to ask you about your exercise habits. Is it okay if I record this interview?

Leo: Yes.

Hanisah: Can you tell me your name?

Leo: Leo Hwee Xien.

Hanisah: Okay, what is physical activity?

Leo: What is physical activity? It's the activity we do to make us sweat, and become healthy.

Hanisah: Okay, what is exercise?

Leo: Exercise ah? Exercise is, uh, any activity also that need…that will like use up our energy.

Hanisah: So, what's the difference between physical activity and exercise? Because it seems the same.

Leo: Physical activity can be one of the exercise.

Hanisah: Okay. Anything else?

Leo: Uh, that's all.

Hanisah: Okay. Um, do you exercise routinely?

Leo: No.

Hanisah: Okay, why don't you exercise?

Leo: Tak suka. Haha

Hanisah: Okay. You tak suka exercise. Can you tell me why?

Leo: Um, I also don't know lah actually. Maybe…maybe no motivation lah. Hahaha

Hanisah: No motivation, okay. Um, what are some of the things that you don't like about exercise?

Leo: After exercise will be very tired and um, time-consuming.

Hanisah: Time-consuming. You don't have enough time in a day to exercise is it?

Leo: Not really not enough time lah but just um, I didn't make the time for it. Hahaha

Hanisah: Okay. Fair enough. Can you tell me for yourself, why do you think exercise would be good for you?

Leo: Because it can make us sweating, and increase our metabolisme in our body, make us feel like fresh after exercise.

Hanisah: Okay, what can you do to start exercising? What can make you feel more motivated to start exercising?

Leo: Maybe if got a companion.

Hanisah: Other than that? What would also make you feel motivated?

Leo: Feel motivated ah? Become healthy lorh.

Hanisah: Um, if…if I have like a program like say for example, I do a program like a free zumba class, would you feel…for example like if I do a program like a free zumba class, for example, you feel you want to join?

Leo: Um, if got time can lah.

Hanisah: Okay. How much time are you willing to spend on exercise in one day?

Leo: 30 minutes to 1 hour.

Hanisah: 30 minutes to 1 hour, okay. What can we do so that you can feel motivated to exercise routinely? Means 2-3 times a week. What can make you feel motivated?

Leo: What can you do ah? Held some activity near to us lah.

Hanisah: If it's something that you do yourself so that you will exercise routinely?

Leo: Tak faham.

Hanisah: I mean like, um, if I give you a guide on how to exercise routinely and then I just tell you, okay, you can do it yourself. What would make you feel motivated so that you will follow the exercise module tu yourself.

Leo: If I know how the exercise activity or the exercise module can benefit me, like what I can get in return, only then am I motivated to do it.

Hanisah: Um, example of the benefit like say if I put, if you do this exercise then you burn this much kilocalories, is it like that?

Leo: Um, can be.

Hanisah: Any other benefit?

Leo: Not really lah.

Hanisah: Okay. What kind of exercise do you like?

Leo: I like water activity.

Hanisah: Something like swimming lah?

Leo: Yes

Hanisah: Other than that? Like something you can do in college lah.

Leo: Cycling can lah.

Hanisah: Cycling. What about naik turun tangga?

Leo: Penat. Hahaha

Hanisah: So you don’t like naik turun tangga?

Leo: No.

Hanisah: Okay, it’s okay. What about jump rope?

Leo: Jump rope ah? Not too much lah. I mean the time.

Hanisah: Okay. Stretching, what about stretching?

Leo: Can, stretching.

Hanisah: Stretching okay.

Leo: Not too much lah. Hahaha

Hanisah: Okay, not too much. When we produce the exercise module, do you want to join?

What would make you want to join?

Leo: I see what is the activity involved in the module. Okay kot.

Hanisah: Okay, so it has to be an interesting module then only you want to join? What do you want in the exercise module?

Leo: Easy! Hahaha. Something like, not difficult to do and can do in room.

Hanisah: Something easy, not so time-consuming, can do yourself in your room. Okay. Um, would you exercise every 2 days? Means you exercise, then you rest 2 days, then you exercise again.

Leo: Can.

Hanisah: It’s okay?

Leo: Yes

Hanisah: Why you say you will exercise every 2 days? Is it convenient?

Leo: …

Hanisah: What would motivate you to exercise that often?

Leo: That often, very often leh? Hahaha, dunno.

Hanisah: It’s quite often compared to now lah. Haha

Leo: Become healthy. The main lah.

Hanisah: Okay. It’s mainly because you want to become healthy. When you exercise what is your goal? Is it to become more fit, is it to lose weight, is it to become more muscle-y, what is it?

Leo: Muscle-y? Hahaha. (joking) Um…(seriously thinking) Lose weight and look fit.

Hanisah: Lose weight, become fit but not muscle, okay. Should we include running in the exercise module?

Leo: No, I hate running.

Hanisah: You hate running? Okay. That’s all we want to ask you.

Transcript Respondent 3
Name: Shakt
Age: 23 years
Faculty/Program/Year: FSK/Biomed/4
Respondent Code: R3

Interviewer: Hello my name is Hanisah. Shakty thank you for talking to us today. Is it okay we interview you and record?

Shakty: Yes. It's okay.

I: Umm. What is physical activity?

S: umm. Physical activity is more like doing exercise using your whole body movement. That all about.

I: Okay. Umm. What is exercise?

S: Exercise is a form of a body movement that urmm in order for us to stay, keep healthy, keep fir and make sure all our body is good, healthy stage.

I: How is exercise different from physical activity?

S: Physical activity is something like we usually do in everyday lifestyle while exercise is something a bit aaa more, slightly vigorous. It not always we do, is more towards we do in order to keep our body fit. Physical activity is more like what we usually do but you also help us to reduce calories.

I: Okay. Umm. Do you exercise regularly?

S: Not often.

I: Okay. Aaa. Why don't you exercise?

S: I have. I do not have much time to do it and got lab work.

I: Ada any other reasons aside from time constraints?

S: Urmmm. I don't think so because of my time constraints and aaa even if I'm okay maybe I won't be in good state, not very healthy maybe I sick.

I: Okay. Umm. What are the thing that you don't like about exercise?

S: What I don't like about exercise. Umm. We need a very open space to do it and aa sometimes certain exercise required equipments like dumbbell or something like that if we don't have you it not have the feeling of your exercising.

I: Umm. In your own opinion what do you think that exercise is good for you?

S: It good for me because if I exercise often I feel much more, I feel much more active, I feel a bit fresh and aaa I feel like I can walk a bit longer time and I can do thing at a long aaa I can do activities much more like actively, not sleepy mood and because of six pax.

I: Okay. Urmm. What are the things that can motive you so that you can start exercising?

S: Urmm. If I have time one and if there anything like aaa a way that I don't need to have any equipment and doesn't require a lot of space. Well because I'm still in a small room so maybe if like in a small space like if I can just sit anywhere and I can do exercise it would be better.

I: Okay. Urmm. If we can achieve all that, would it make you exercise routinely and why?

S: Urmm. Probably I will make it routinely if I have that kind of way because well is better to be exercising and keep your body in a perfect healthy state. Because if we. I can see the differences when I didn't exercise. When I don't exercise I feel very, my body aches.

I: So aa you will be more motivates to exercise routinely if you can become more healthy. You can achieve this goal. Like thatlah. Okay. What kind of exercise to you like?

S: What kind of exercise. Aaa. I prefer more towards the lower abdomen part of the exercise. Urmm but for me I need to workout my upper abdomen exercise.

I: Okay.

S: I more prefer like kind sit up. Aaa no. Sit ups and more towards legs, that require lots of movement in my legs.

I: Urmm. What about going up and down the stairs? Stretching?

S: Aaa. Going up and down the stairs?. I hate going up and down the stairs but its very good also because aaa it strengthens more. I like stretching because it makes body move flexible and also reduces the gases in our body.

I: I see. Urmm. What about running? Do you like running?

S: Yes. I love running.

I: Okay. That's good to know. Aaa. If we. We will produce an exercise module for female KTSN students would you join and what would make you join?

S: Urmm. Probably I would join. Aaam. What would make me want to join probably because I need to work out plus urmm I really umm stop working out ever since I not participating sports so yeah because I feel that working out I body is much more better conditioned.

I: urmm. What do you want in an exercise module?

S: What I want? Urm. Exercise module, I feel like an exercise module should have like that covers the whole body from the lower abdomen to the upper abdomen, the hands everything. Urmm. Like it should be balanced and if possible something that requires urm no equipments and you can do it in a room. In a small area of space.

I: Okay. Urmm. Would you exercise every two days? Like you exercise then you take 2 days break and then you exercise again?

S: Ummm. Sometimes yea. It depends

I: Time?

S: My body and yea nothing.

I: Okay. What would motive you to exercise that often?

S: Ummm. What motives me?

I: What motives you?

I: It could be friend or you have a goal to keep. Whether you want to stay healthy or because you want to get out with your friends?

S: What motives me. Probably because I'm. If I could stay keep healthy and if possible, join any like sports events.

I: okay. What is your goal when exercising? If you want to get more fit, lose weight, build muscle. What kind of goal do you have?

S: My goal is mainly I want to do, if possible six pax and mostly build muscle. In terms of losing weight I think I will lose it automatically if I exercise. So,my main goal would be build muscle and make myself fit.

I: Okay. Thank you.

Additional Questions

I: What type of intensity do you prefer when you exercise? Light, moderate or vigorous?

S: I like vigorous exercise more.

I: In a week, how many days would you be willing to exercise?

S: Now, 1 day or 2 days.

I: In your opinion, how long should the duration of the exercise module be?

S: Exercise module, aahh, I'm not sure about the duration like how many hours in a day or how many days in a week we should exercise.

I: What I mean by duration is, in total, how long would you like the exercise programme to be? Maybe a month or 2 months, like that.

S: Hmm. The duration should depend on the goal of us exercising. If we are training for sports, the programme should be a year. If to lose weight, 2 months depending on the type of exercise done. If based on your experiment, results will only show after a month.

I: In a day, how long would you prefer to spend on exercise?

S: I think 30 or 40 minutes.

Transcript Respondent 4
Name: Hanisa
Age: 23 years
Faculty/Program/Year: FSK/Biomed/4
Respondent Code: R4

Interviewer: Assalamualaikum Hanisah. I'm going to ask a few questions about exercise. Would it be okay if this interview was recorded?

Respondent: Sure. Sure.

I: Okay. Aaa. Do you exercise routinely?

S: No.

I: No. Okay. What do you understand about physical activity?

S: Physical activity is a, any movement that involves the bones and muscles.

I: Okay. What do you understand about exercise?

S: Exercise is one of the physical activities that we do. There are specific things that we can do.

I: Okay. Is there a difference between physical activity and exercise?

S: Physical activity. Exercise is a type of physical activity.

I: Hanisa, aaa, is there any reason why you don't exercise?

S: Because, ummm, laziness probably, because of myself.

I: Does laziness mean you don't feel motivated to exercise?

S: Yeah, no motivation. Sometimes I do but I'm just staying in my room, relaxing. In the end, it's suddenly morning or noon and by then I have other things I need to do.

I: What is it that you dislike about exercise?

S: Hmmm. I'd feel a bit tired after.

I: Is there anything else other than that?

S: hmmm

I: Maybe because of sweat? Or maybe there is a lack of space?

S: No.

I: No. Okay. Aaaa. What do you think are the benefits of exercise for yourself?

S: Aaa. There are lots. Of benefits, I mean. Firstly it will, aaa, we can sweat. So, when we sweat, we will feel more energetic than normal. When we study after that, we will be more focused compared to when we don't exercise at all.

I: Is there anything else?

S: Hmm. I think that's it.

I: That’s it? Okay. Umm. If you were to start exercising, what would make you want to start?

S: Aaa. Free time.

I: Other than that?

S: Umm. Motivasion.

I: Self-motivation?

S: Haah. Right.

I: What about a goal?

S: Hmmm. I don’t really have a goal.

I: If you had started exercising, what would keep you motivated to continue exercising? Keep at it, I mean.

S: Hmm. If it was fun.

I: Fun. Is there ummm..

S: What I mean is that when we have started to exercise, our bodies will feel more energized. Like that lah. Feeling that way would make me want to exercise again.

I: Basically aaa you like the rewards from exercising?

S: Yeah.

I: Okay. What type of exercise do you like?

S: Ummm. Type of exercise. There isn’t a lot that I like honestly. In fact, I don’t think there’s any particular one that I like.

I: Aaa. How about swimming?

S: Umm. Not really.

I: What about dancing?

S: Hmm. Not dancing.

I: How about a walk?

S: Walking is okay. Brisk walking or playing football or something like that is okay.

I: Football aahh. What about skipping rope?

S: I don’t like it that much.

I: Aaa. Stretching?

S: Not really.

I: I see. What about going up and down the stairs?

S: Not interested.

I: Ooo. Not interested.

S: I don't know. I think I like sports more. Even if it's jogging, it's not that I'm interested in it. It's just that because I know the benefits of jogging so I try, even a little, even if sometimes I feel tired, I'll still try. Okay today, I'll jog for just 10 minutes, and maybe tomorrow I'll jog for a little longer.

I: Okay. Aaa. If we were to design an exercise module, what would make you want to join the exercise programme?

S: Exercise module? Hmmmm. Aaaa. The exercise programme shouldn't take too much time. The duration should be just nice.

I: Just nice is like 30 minutes?

S: Hmm.

I: How about an hour?

S: Haaa.

I: Is 30 minutes okay?

S: Yeah. 30 minutes.

I: What kind of exercise should be in the module? Something interesting, or maybe something challenging?

S: What's an example of something interesting?

I: Something interesting would be not just sticking to one type of exercise but rather have a variety. Today do one type of exercise, tomorrow do another type of exercise.

S: Then maybe something interesting. That type.

I: Variety huh?. Aaa. Would you be okay with exercising 2 alternate days in a week?

S: Hrmm. If it's alternating, hmmm. I'll only join your exercise programme if it's in the morning because I prefer to exercise in the morning. If it's in the morning and doesn't clash with anything, then, I'm okay.

I: Okay. If we do the exercise module in a book where you can do it whenever it suits your schedule, would you be interested?

S: Hrmm. I'd need motivation.

I: You'd need motivation Okay. Aaa. What would keep you motivated to exercise on alternating days?

S: Huh.

I: For example, if you had a friend come with you or you had a goal you wanted to achieve with the exercise.

S: Having friends does play a role. If I didn't have a friend, and I had to do it alone, I'd feel like nah Nevermind. If I had a friend who was excited to join, likes sports, they would help motivate me a little. One of the factors lah.

I: What other factors are there?

S: Aaaa. I'm not sure.

I: What about time or sports equipment?

S: Ummm. Time. If it’s time, like I said just now, I don’t want it to take a lot of time. It also depends on the day, maybe some days I’ll do it in the mornings if I have nothing else to do. If I’m busy, then I won’t exercise.

I: What about setting a goal?

S: Not a goal. What’s an example of a goal?

I: An example would be maybe to take care of your physical appearance, or your health, or to lose weight.

S: Hmmm. In terms of health, yeah, it does motivate me sometimes. Fore example, feeling healthy, feeling energized etc. It does motivate me a bit.

I: Ah, it motivates you a bit.

S: Yeah, a bit but not to a significant extent. Not that much really. It’s more like it influences me psychologically, my mind set. What I want. Just in case if I wanted to do an intervention using this exercise module, I’d have to properly fix my mind set on why I’d want to exercise.

I: Okay, that’s all. Is there anything you would like to add?

S: No.

I: Thank you.

Transcript Respondent 5
Name: Faliha
Age: 23 years
Faculty/Program/Year: FSK/Nutri/4
Respondent Code: R5

Hanisah: Okay, this is Hanisah. Can you please give me your name?

Faliha: Faliha.

Hanisah: Okay, Faliha. Um, what is physical activity in your opinion?

Faliha: Physical activity is when we start to moves, means there is sweating at least in 10 minutes or 30 minutes lah.

Hanisah: What is the meaning of exercise?

Faliha: Exercise…involving body movements or something like that lah.

Hanisah: What is the difference between physical activity and exercise?

Faliha: Oh. Oh, no. I think physical activity is which involves our movements. Exercise is like we are..are..meaning that we already produce sweat or something like that such as berlari.

Hanisah: Okay, it's okay. Um, you said you dont exercise regularly. Why?

Faliha: ah, first of, lazy. Actually got do also but most importantly lazy, lack of facilities for exercise, but mainly lazy lah.

Hanisah: Can we say that every week you will exercise at least once?

Faliha: Ah, yeah. I got exercise, weekend lah normally. in my room…

Hanisah: Okay lah, good. Um, what are the things which you do not like about exercise?

Faliha: Exercise…first first, when exercising outdoors, I feel a bit ashamed when exercising with people who are already fit and I, on the other hand, am not.

Hanisah: Okay, um, are you feeling shy about body shape?

Faliha: uh, I am feeling shy on my height only. Hahaha

Hanisah: Okay, um, what do you feel that exercise is good for yourself?

Faliha: If when doing exercise, it feels more fresh, like feeling better lah for doing work. Feeling not easy to be tired.

Hanisah: Does exercising make you happy?

Faliha: Ya, sometimes exercise makes me happy. Feel happy lah especially when we enjoy the exercise.

Hanisah: Okay, um, what can makes you start exercise?

Faliha: Start exercise, because for example like exam season, eat a lot right? especially at night, so weight will increase. So normally these kind of things makes us want to exercise.

Hanisah: um, what are the things yang that can makes you exercise regularly? Like you continue exercise not start only.

Faliha: Normally because of feeling fun as I always do aerobics, when we feels fun when watching the video, and then watching their fit bodies, oh, its better that we can follow and be like them. At least we tried to become like them right?

Hanisah: You like zumba?

Faliha: Ya, I like zumba hahaha

Hanisah: Okay, um, except from zumba, what else exercise that you like?

Faliha: I like playing handball, um, badminton. Even not good at playing but its is fun plus we are playing with our friends right.

Hanisah: If the type of exercise alone which involves stretching, up and down stairs?

Faliha: Up and down stairs! Because my room is at higher floor, and will think everytime that this is actually exercise for up and down stairs.

Hanisah: Okay, um, if we do one exercise module for students, do you want to join? And if you want to join, why?

Faliha: Ah, if interesting, like different from the others, InsyaAllah can join. Ah, like I said, if the module is an interesting module and not difficult to be carried out.

Hanisah: What do you want to be included in this module?

Faliha: The module? like I said just now, like Zumba, so, basically which includes music, so, more interesting lah.

Hanisah: If the exercise module asks you to exercise alone in the room, you want?

Faliha: Ya. If sometimes got people ask to do then monitor, that is the kind of things that makes us feeling more likely to do compared to doing ourselves. Sometimes we will feel lazy right? If got people want to monitor, later they would want to see the result so we will do lah.

Hanisah: Okay, um, if we ask you to exercise every two days, meaning that you exercise, you rest two days then you exercise, do you want it?

Faliha: Okay. Okay je. Because actually we need to exercise daily right?

Hanisah: Okay, ah, Can we include running in the module?

Faliha: Can, but like when in the beginning must not be too long, nearer like that.

Hanisah: Oh, so you want something which is easy to be done?

Faliha: Ya, the starting must be easier, later if progress to a later stage want to be more difficult that is okay lah.

Hanisah: Okay, if you exercise, what is the goal which you want to achieve? Get fit, build muscle, ah, lose weight, or others?

Faliha: I want to be fit because I used to play handball a lot and back then, my body felt so great. I don’t know how to explain it but it’s great. When I wake up I feel great but now that I don’t play as much, I get tired easily. So to be fit again would be great.

Hanisah: Okay, thank you.

Transcript Respondent 6
Name: Sulima
Age: 26 years
Faculty/Program/Year: FSK/Biomed/4
Respondent Code: R6

Interviewer: Hello, Puteri. My name is Hanisah Hossain. I'm doing a study on exercise patterns for female students in KTSN. We want to make an exercise module so female students in KTSN are more active. This interview will be recorded. Can we start? Um, do you exercise routinely?

Respondent: Right now, no, not routinely.

I: How about we include the last 2 weeks, for example.

S: Last two weeks, uh, no. Not really. I haven't really exercised, and not routinely. I just take walks but not exercise like jogging or anything like that.

I: Oh, okaylah, not routinely. Okay, if it's like that, what do you understand about physical activity?

S: Physical activity, are activities that uh, require movement of the whole body and requires us to sweat. Something that requires energy.

I: Is there anything else that you think defines physical activity?

S: Any daily activities that we do, even walking is a physical activity. Lifting weights, bringing bags, at least I think so. Walking, jogging.

I: Ah, it's a wide scope then.

S: Ha, yes, a wide scope.

I: What do you understand about exercise?

S: Exercise, exercise is, uh, more to doing physical exercise that really makes you sweat. How do I explain it? More ahhh...

I: More energetic?

S: Yes, exercise makes us more energized, and sweat more.

I: What is the difference between physical activity and exercise? In your opinion.

S: In my opinion, physical activity is a general thing like daily activities are counted as physical activities but exercise is more like, how do I say this? An activity that really requires our body to work hard and sweat and use all the muscles that we have. Hahaha.

I: Like a program?

S: Ah, program ha. It's easier to do vigorous activities.

I: Oh, vigorous?

S: Ah, vigorous.

I: Oh, okay okay. Um, if you don't do daily exercise, why do you feel that you don't want to exercise?

S: Um, it's not that I don't want to exercise, ah, but, due to time restraints and lack of space or planning lah. Sometimes we don't plan. Usually, when it comes to exercise, when you plan, you end up doing it. You have the feeling of doing it, and then when you initiate it, there's definitely something that stops me.

I: Is it the lack of motivation? Something like that?

S: No motivation? Ah, I do have motivation but I feel like it disappears quickly.

I: Oh, disappears quickly. Okay, um, is there…like say you have a lack of space for example, ahh, is there a lack of space to exercise in KTSN?

S: Oh, eh. There is. Inside the college, like at KTSN, there are programs where you can exercise like the Taekwando club. And then other than that, on the outside there's also silat, right? Outside the college, like at the Titiwangsa Lake, there are places to exercise where we can jog a round or two or the exercise at the spot with built-in equipment.

I: Do you exercise at the lake?

S: Exercise: I've exercised there many times. Jogging...usually, but not now though, but I used to.

I: I see, you did?

S: Haah. Yes.

I: What about at Plaza Rah?

S: Plaza Rah….I do, mm? Swimming because there is a swimming pool so that's why I can. If I don't go to the lake, I'd definitely go swimming.

I: But the pool is only for Plaza Rah residents?

S: Yes, only Plaza Rah residents. There's a gym in KTSN as well if I wanted to use it.

I: Ah, Is there anything that you dislike about exercise?

S: The thing about exercise that I don't like is that after exercise, especially when the body is just beginning to adapt to exercising, I'll get body aches and pain after. In other matters, at least for me, my face gets all red, and then if I sweat, my face would get so red because the blood would rush to my face. Haha.

I: Do you feel any discomfort when people can see you exercise?

S: Uh, it's a problem when I wear normal clothes but when I move around, it can accidentally lift up and my body is exposed. If I see males around or anything, I'd definitely feel a bit shy.

I: Oh

S: On top of that, I’ve never really gone to the gym because if you look at the gym, there are usually a lot of men in there. I know that some days are designated for women but when you have to share, I haven’t gone. Even when I jog, of course you have to cover up a little bit right? Cover up what needs to be covered up.

I: and then do these things demotivate you to exercise? Are they factors?

S: They aren’t a factor, but it also depends on the situation. Sometimes we accidentally find ourselves in these situations because sometimes you aren’t alone when you exercise or our hijab moves or the wind moves our clothes around. Something like that.

I: Um, do you ever feel like you don’t want to exercise due to body size? Maybe you might feel like you aren’t fit or anything like that? Do you?

S: Um, about body size, I actually, it motivates me more because I know what my body size is like. Actually I become more motivated to exercise. It doesn’t make me give up on exercise but if a person is already fit, and maintains their fitness, or if a person who definitely needs to exercise, I think they would definitely have the motivation to exercise. I don’t think that they would lose their motivation to exercise.

I: Okay, Um, what do you think are the benefits of exercise for you?

S: Basically exercise is good to lose weight, burn calories, and detoxing through sweat. What’s important is that, my body will be more fit, and I can feel more energized. It would be easy to climb up and down the stairs without feeling tired.

I: um, in your life, what can someone else do (us) to make you more motivated to exercise?

S: Like, somebody else do eh? If there was a step-step, uh? Like, what? Stes to do the exercise, or uh, yeah steps I guess. A program that can, or, uh, there was an exercise program in KTSN the other day but I didn’t join. Something like if it was a zumba event, I’m okay to join.

I: So, if there was an event lah?

S: Yeah. If there was an event or a coach. It would be easier. Like back then, taekwando was easy because there was a coach so I just had to follow him.

I: aah, I see.

S: I want to exercise more if there is someone at the front to teach me.

I: But say if you were alone, is there anything that we can change to motivate you to exercise?

S: Maybe if I could be friends with someone who knew about exercise or somebody else who knows how to exercise, I would.

I: Okay, um, for example, if we did an exercise module especially for female students living in KTSN do you think you would try the exercises?

S: um, sure if I had the time.

I: Ah, okaylah. So, there’s a chance? Haha

S: Yeah, if it was interesting.

I: Okay, what would help motivate you to continue exercising?

S: To continue exercising?

I: Like say you have started trying to exercise, what would keep you motivated?

S: When there is a change in my lifestyle like when I’m not even tired after going up and down the stairs. My body feels more fit and I will definitely feel more addicted to exercise. To me, I’m hooked. For example, with tae-kwando, my body only hurts for 2 weeks and then we start with movement week, it’s great. Just great.

I: Oh, if you have set a goal, then you have achieved that goal, and then you feel better lah?

S: Yeah.

I: Okay, what type of exercise do you prefer?

S: Um, stretching, jogging, swimming. Ha, those.

I: Stretching, jogging, what about going up and down the stairs?

S: Yeah, going up and down the stairs. That’s even better.

I: That’s even better?

S: Yeah. Like, before this, when I was at the hospital, I definitely needed to climb up and down the stairs. I didn’t feel that tired. Most people prefer to use the lift, right? I like to use the stairs hahaha. Like even at Plaza Rah, the only problem is that the stairs are inaccessible. If not it would be much easier to use the stairs.

I: What about jump rope? Skipping rope?

S: That’s okay too. I do have a skipping rope, if I go to the lake, I’d usually, definitely bring the skipping rope.

I: It is your own?

S: Yeah. It’s mine.

I: What about running? Like say a 10 second sprint.

S: Back when I joined taekwondo, I did have to do that. There was also jogging followed by sprinting then we could slow down a bit.

I: So do you like to do it?

S: I had to. Haha because I'd already joined the program and there is someone teaching me, right? So, I definitely had to do it, if not I'll definitely get it bad.

I: Okay, um, if we do an exercise module and make it like a program, for 2 months for example, and then we personally look at your changes, look at your progress, personalize the exercise for you, would you like to join?

S: I think I want to because I think based on my necessity, I will look at what I can achieve, my goals, maybe I would like to join.

I: What would attract you to join?

S: If there was dietary/ nutritional advice as well.

I: Dietary/ nutritional advice?

S: Yeah because sometimes we exercise but we can't control our food intake. I'm a bit weak-willed when people give me food.

I: For example, we give you an exercise routine, then we calculate how much kilocalories it can burn, would it make you more interested in exercise?

S: Yeah, I'd be more interested. Like, then I'd know how much I can burn from that exercise routine. So, that's better.

I: If we don't give you that incentive, would you still be interested to join for 2 months?

S: Because it's for my own good, I think I'm okay to join because at the end of the day, I'm the one who benefits.

I: But, say we were to give some sort of incentive to exercise, what would you be interested to receive?

S: Um, maybe some healthy food.

I: Healthy food?

S: Yes. To do the exercise, like a chocolate bar. Something healthy like a granola bar.

I: How about fruits?

S: Yeah. Um, maybe an energy drink, or something like that lah.

I: Interesting, okay. Uh, okay. What would keep you motivated to exercise, for example we give you an exercise routine that alternates between 2 days. You exercise for a day then you recover the next day, then you exercise the day after recovering.

S: There has to be, uh, what? A plan lah, like, if it fits in my daily schedule, then I'm okay with it. What's important is that there is determination to exercise.

I: So, you want an exercise program that doesn't take a long time during each session?

S: Yeah, when I want to exercise, it does depend on the timing of my schedule. Maybe at any certain day, I can allocate some time, say maybe half an hour or an hour or so.

I: mm, if we give you an exercise program for you to do by yourself, do you think you would do it and on alternating days?

S: I think I'm okay to do it if I already know what to do and what the steps are. I could definitely do it within my capabilities, I would.

I: Okay, okay. That's all we wanted to ask you. Thank you for joining this interview. Okay, thank you, Puteri.

S: Okay, your welcome.

Transcript Respondent 7
Name: Zuraini
Age: 27 years
Faculty/Program/Year: FSK/Biomed/4
Respondent Code: R7

Interviewer: Do you exercise routinely?

Respondent: Yes.

I: Yes. What do you understand about physical activity?

S: Aaa. Physical activity is any movement, ahh, that uses skeletal muscles, and requires energy lah.

I: Is that all that you understand from it?

S: Yes.

I: Aa. What do you understand about exercise?

S: Exercise huh? aaa. Exercise is one of the types of physical activities, aaa, and exercise is a routine that is structured. Meaning that there are organized modules available, not just something that you can just wing it and do.

I: Is that all that it means?

S: Yeah.

I: When did you begin exercising routinely and what are the reasons you started?

S: Aaa. I started aaa, uhh, exercising around 3 or 4 months ago. I started because I had a change in address where my current accommodation provides access to a swimming pool for its residents. So, I can always use it at any time, depending on when it's open but I usually use it during the weekends.

I: Aaa. Why is that?

S: That's because when I exercise, by swimming, I feel like I can swim for a long time. When exercising, I feel like I need a high amount of stamina. If I swim for 2 hours per session then I can swim for the whole 2 hours non-stop.

I: Aaa. How many times do you exercise in a week?

S: Aaa.. I don't have exactly a set number of days that I exercise. I only exercise whenI have spare time. So, there isn't any fixed amount of days in a week, eventhough WHO recommends 3 times a week but I only exercise when I have extra time.

I: If you have extra time, how long is each exercise session?

S: Aaa. Each session that I usually do, if I swim, because I choose swimming as my activity, I'd usually swim for 2 hours. Say I start at 12 in the afternoon, I'd finish at 2pm. uum. Usually on the weekends lah.

I: Weekends?

S: Hrrm, yes.

I: 2 times?

S: Two times a week or if on a normal day, if I have time, yeah, I'll exercise.

I: Do you feel that it is enough for you?

S: Aaa. Of course not because it doesn't meet the conditions of WHO recommendations.

I: What helps you to stay motivated to exercise?

S: Aaaaam. When. When at the end of the month, I check my weight on a scale and I can see that there is a decrease. Yes, I feel very happy because that makes me more enthusiastic to continue exercising.

I: Aaa. So, your motivation, aaa, results from doing the exercise, is it?

S: Yes. Yes, aside from exercise, I do diet as well.

I: What are the barriers that stop you from exercising?

S: Aaaa. Yeah. This first one is this lah, a busy schedule, if I have a full day of class on that day, then I definitely won't exercise. If I have spare time in the morning for example, InsyaAllah, I will do it but if, usually lah, if I have class in the afternoon then I won't exercise. I definitely don't have time anyways.

I: So it is like time constraints?

S: I have too many barriers actually that make it hard to exercise routinely.

I: In your opinion, what are the benefits of exercising for yourself?

S: Aaam. Exercise, um, in my opinion lah, what I can remember. Benefits. An example of benefits would definitely be that it can increase my stamina. Aaaaa, aside from that, uuurm, it can also increase my physical fitness lah. In terms of my own fitness, in order to maintain, aaa, one and the main thing is to lose weight and the second is to increase my stamina.

I: So, aaa, the benefits of exercise are only weight loss and stamina, right?

S: Yes.

I: If our exercise module requires you to exercise on alternating days, would you be okay with that?

S: Aaa. It depends on the module itself, if the exercise module recommends how many, aaa, for example it involves cardio, and it also depends on the duration of time each exercise session will take to do.

I: Say if the duration of time is more or less 10 minutes, would you be okay to do it?

S: Yes. InsyaAllah. If it is 10 minutes.

I: What about an hour?

S: If it's an hour, it depends aaa, if we want the exercise to be more effective, we need friends. If we are alone, usually motivation is only there for a brief time. Motivation is only here for a while. It actually needs, if you do this exercise program in a group, I think this module can succeed.

I: Okay. So, it depends on the exercise module itself?

S: Yes.

I: Aaaa. What would keep you motivated to continue exercising on alternating days?

S: You mean, if this module is executed or what?

I: Yes, if this module is executed.

S: Aaaa. If this module is executed, it can aaa, help me, aaa, achieve my stamina and also weight loss and I think that I will do it.

I: What about outside factors, like motivation from friends maybe or the environment maybe?

S: Yes. That also plays a role.

I: What if, say for example that the exercise itself, in the module, is easy to do?

S: Yes, if the module is easy to follow. It doesn't need what some people say are difficult steps (in the exercise module) for the people who want to join, ahh. If it's easy, I think there won't be a problem. I think if this module was in video form, it would be better.

I: What type of exercise do you prefer?

S: Because now I, uhhh, often go swimming, so, for now, my favourite activity is swimming.

I: What are the things that you dislike about exercise?

S: Aaaa. The thing I dislike the most when exercising is when I sweat. Because, yeah, sweat.

I: Sweat.

S: If I swim, I don't sweat because I will already definitely get fully submerged in the water.

I: Aaa. Umm. What is it about sweat that you dislike?

S: Aaam, sweat actually makes me feel uncomfortable.

I: Uncomfortable, huh?

S: Yeah.

I: Okay. We, we now have oyur input on swimming. What other exercise activities do you like?

S: Other than my favourite activity which is swimming, I also like zumba because zumba is easy to follow. This is because we only need to look at the video and we can do it alone, solo.

I: Something like dance then?

S: Dance, yeah.

I: Alright, that's all the question. Thank you so much.

S: Your welcome.

Extra questions

I: In the exercise module, what type of exercise intensity do you prefer? Light, moderate or vigorous intensity?

S: Moderate intensity, alternating with vigorous.

I: in a week, how many days are you willing to exercise and how long would you exercise for each session?

S: I’d exercise for 2 to 3 times a week and around 30 to 60 minutes per session.

I: If I were to do an exercise module for you, approximately how long, I mean, how many months would you like the exercise module to be?

S: 3 months.

Transcript Respondent 8
Name: Norfathin
Age: 23 years
Faculty/Program/Year: FSK/Biomed/4
Respondent Code: R8

I: Oka. I will be asking a few questions regarding exercise activities, exercise activities ya. This session will be recorded. Is that okay?

S: Okay. You can

I: Okay. The question, the question, aaa do you do exercise activities routinely? Like doing it regularly

S: I have

I: Okay. What do you understand about physical activities?

S: Exercise activities that we do, body movements for health benefits, more healthy. Exercise is like jogging, aerobic exercise, things that make you sweat.

I: What do you understand about exercise?

S: Exercise are physical activities that we do routinely, I a week, we do 2 times, 3 times, like that, according to our free time, for health purposes.

I: What uis the difference between both

S: The difference between physical activities and exercise. We do exercise routinely. Physical activities, we do everyday like writing, it's also a physical activity.

I: What makes you start exercising?

S: Makes me..

I: Routinely

S: Aaa. What makes me. Because I want to release stress, so I do it

I: When do you start the exercise?

S: When? When I feel I need to stop doing work such as assignments. When I feel exhausted, need to stop, usually during the Asr prayer time. After Asr, I would do exercise.

I : I meant when, when do you really do it routinely? The past one month, or 2 months?

S: Ooo. It has been a while. How do I remember?

I: give me estimates

S: Aaaa. A few months ago, a year, maybe

I: A year

S: maybe 2 years, 3 years

I: 2 years, 3 years

S: I'm not sure, Over that, started when I was in 3rd year

I: 3rd year. About a year ago

S: Haa. A year. Yes. A year ago

I: how often do you exercise, like how many times in a week?

S: 2 times a week, 3 times

I: 2 to 3 timnes a week. Okay. Do you think that is enough?

S: No

I: No

S: Not enough

I: Why?

S: Because when we're doing aerobic exercise in a short period of time, in 30 minutes. It's tiring, really tiring. We sweat, sweat but at the end, we go and eat. After eating, we feed, then we rest, then there's nogt much time. 30 minutes is a short time. 30 minutes is not enough. Not enough.

I: Not enough duration, it seems

S: Hmmm. Not enough

I: What would help you to stay exercising?

S: Why? Eh, What could help?

I: Like if you have any goal or..

S: Haa

I: If you have friends or goals?

S: Aaa, Goal

I: What is the goal?

S: The goal is like, I actually would like to eat according to the recommended calories, so I try to exercise to complement the limit, for example my daily limit is 1200. So my target by the end of the month is to lose 1 kg. 1 kg or…

I: So this is for body appearance, you exercise. To reduce weight

S: Not so much on body appearance. The other day, I did a check-up and my body fat is higher than normal, so I have to do it

I: So you are worried about your health

S: Haa. Yes. Exactly

I: Apart from that, is there any reason?

S: Hrmm.. mostly. No Servik (Inaudible). That's why I want to reduce body fat

I: Fat content. Don't you feel happy when you exercvse?

S: Release stress

I: Release stress

S: Yes. It’s fun

I: What would hinder you from exercising 2 times every 2 weeks

S: 2 times a week

I: 2 times a week. What hinders you from doing it 5 times a day?

S: Hinrance. Firstly because I have already set the limit to 2 times a week, I target to this slowly, I do not want to go to the extreme too fast, it might affect mny body. Don’ty want to shock the body. Well, not that shock because I’m used to it. 2 times a week is plenty enough. Also because of time factor. To do assignments.

I: So it’s about time. You have other work to do

S: Yes.yes. most of it

I: Is there anything else? Maybe no designated space?

S: No designated space. You can do aerobics anywhere. But it’s probably right, if there is a women only gym, I would go

I: Or you probably need friends?

S: Haa. Umm. With friends, we can do much more

I: Okay

S: It’s no fun doing alone

I: How about needing specific equipments?

S: No. No. that is not required

I: Aaa, when you do aerobics, what do you get from it?

S: aaa. Satisfaction. And when we are stressed, we feel so refreshed. From being stressed, then after we finished doing it, yes we can, we end up being satisfied. Then we sweat a lot. The body feels lighter and less pressure. We feel stressed, so the load become lighter. It’s fun

I: Fun?

S: Enjoyment

I: Aaa. For health or for your own self

S: Not sure about my health. Need to check every month. If we go to the doctor

I:How about the feeling of, like, improved health, or become heal;thy, or heart rate, protection from obesity, like that?

S: Aaa. Yes, the obesity stuff. Prevent obesity

I: Diabetes

S: Diabetes is probably more related to food. Not sure about exercise. I have a friend who exercise a lot, but because she did not control her food intake, she got diabetes. She has diabetes, so it's not necessarily…

I: Not necassarily

S: Not necessarily. Food. Her dietary intake

I: Okay. Is that all? Do you want to add more about the benefits?

S: Benefits of aerobics? Yes, that's it

I: That's it. Aaa. If I were to make an exercise module, what does it take for you to exercise today, rest tomorrow and exercise again. Would you do it?

S: Okay. Yes. It's a good thing

I: What about it that attract you to do it?

S: If it produces results such as weight loss, then it's okay. It's very interesting

I: Very interesting. Apart from that, is it because it is interesting, or fun or challenging?

S: Aaa. Fun things are okay. Challenging, not so much.

I: What would make you feel it's fun?

S: It depends on what you ask me to do. If it involves weight liftings, I do not want it. Not my, out of my league. I like things with music

I: Things with music

S: yes. If it's quiet, then shout 1,2,3. I don't want. With music, I will feel excited to exercise

I: Okay

S: One more thing, if we are doing it with friends. It is more fun

I: Do it in group

S: Not fun doing it alone

I: If you do it once every two days. What would make you want to continue?

S: Continue doing it. If anyone invite me, I will join. That's what make me want to do it. If there's nobody inviting, even though I want to do it, maybe not so much

I: Not so much

S: When there's support, we will do it together

I What don't you like about exercise?

S: Aaa. Muscular ache. Leg pain. Body ache

I: Aches

S: Aches

I: Not because of sweating or..

S: Oii. Sweating is good. Sweating make me feel satisfied

I: No other reasons?

S: No

I: No. Is it because it’s tiring or taking oo much time?

S: No. Not really

I: Not really. Aaaa. Okay. Now we know your exercise. What do you like apart from aerobics?

S: Other exercise activities. jogging

I: Other than.. how’s going up and down thw stairs?

S:Ohh. I like

I: Like. How aboue rope skipping?

S: No, just average

I: Just average

S: Less. Neutral. Neutral

I: Neutral

S: Yes

I: How about sit ups?

S: Sit up, yes. I quite like it

I: Pumping

S: Pumping. I cannot stand it. I can do it for a short while only. Because it’s painful

I: So you don’t like

S: I actually usually do it, after aerobics, I would do pumping, but not too long, and not too much

I: Okay, it’s fine

S: Neutral

I: Ok, that’s it from us. Thank you

S: Okay. You’re welcome

Extra Questions

I: In the exercise module, do you prefer light, moderate or vigorous intensity exercise?

S: Moderate

I: In one week, how many days are you willing to exercise and the duration?

S: One week, every day is fine. If I have time, one hour a day.

I: If I’m making the exercise module for you, how long, I mean how many months, that will be included in the module?

S: One month

Transcript Respondent 9
Name: Siti Fatin
Age: 21 years
Faculty/Program/Year: FGG/Pergigian/2
Respondent Code: R9

Hanisah: Okay, I am Hanisah. Can you give me your name?

Nurain: Siti Fatin Nurain

Hanisah: Do you exercise regularly? Like 2-3 times a week

Nurain: Yes

Hanisah: You do? Usually, in a week, how many times do you exercise?

Nurain: I think 2 times

Hanisah: Okay, um, what do you think is the meaning of physical activities?

Nurain: To maintain health's body? That's what I think. And improve our appearance. And to release stress

Hanisah: Okay. What is exercise?

Nurain: Exercise is a physical that ah, improve the fitness or wellness, that's what I think.

Hanisah: What do you think is the differences between physical activities and exercise? Or is it the same?

Nurain: I think it's the same because physical activities can make us exercise. For example, walkinh

Hanisah: Okay. Um, what motivates you to exercise routinely?

Nurain: I think because of my appearance, ah gain weight. Then, I will, uh , exercise

Hanisah: Apart from for appearance, are there any reasons?

Nurain: Uh, toi maintainhealthy body, like when I'm lethargic, ah I exercise.

Hanisah: Okay, do you think the amount of exercise you do is enough for you? Do you exercise enough?

Nurain: I think no

Hanisah: Okay, it's okay. When.. you are busy as a dental student, right. What would motivate you to continue exercise? So that you do not give up?

Nurain: Maybe, my main reason is, uh, to keep healthy lifestyle. Eventhiugh near, uh, we're busy, but it doesn't mean that busy in an unhealthy way.um, maybe we can walk, go up the stairs, go down the stairs, those are okay enough

Hanisah: Okay. What are the things that.. it's the same.. wait, wait… What are the things, for you, that sometimes make you want to give up exercising? Sometimes, you think that you do not like things about exercise. For example you do not like sweating or maybe you do not like people watch you exercise maybe

Nurain: Uh, probably time limit. Ah, because I'm tired from other things, like I cannot exercise enough. For example, like sitting in the room, sleep or do anything else.

Hanisah: Okay. For you, is exercise good for you?

Nurain: Uh, of course. Hahaha, to maintain healthy lifestyle, healthy life

Hanisah: Does exercise make you happy?

Nurain: Uh, seldom. Because even without exercise, we can be happy

Hanisah: I am making an exercise module for female students at the college, if the exercise module requires you to exercise every 2 days, meaning you exercise, you rest then you exercise again, do you want it?

Nurain: Uh, when will it be?

Hanisah: For example, you imagine this exercise module is something that you need to spend about 20-30 minutes exercising and then you exercise on your own, without equipment, do you want to do it?

Nurain: Sure. Uh, why?

Nurain: So that you can be disciplined in exercise

Hanisah: Okay. What kind of exercise do you like?

Nujrain: I like netball, netball, I like jogging, cycling

Hanisah: If the exercise includes stretching, going up and down the stairs, would you like it?

Nurain: Ah yes. For example, my favourite is crunches.

Hanisah: Oh okay. How about Zumba?

Nurain: No

Hanisah: Okay. Apart from exercise, what other physical activities that you like?

Nujrain: Uh, for example. I don't know. Hahahah. Ah, don't know. Maybe walking. Ha, walking

Hanisah. If in the exercise module we include running, like you need to run fo 5 minutes, ah, would you like it?

Nurain: Maybe 2 times a week is okay. If more than that, I probably won’t do. Haha. Due to time constraint.

Hanisah: So you do not want to include running in the module?

Nurain: Um, no

Hanisah: What is…um, this is the last question,what goal do you want to achieve when exercising? Do you want to lose weight, or build muscle, or get fit?

Nurain: All. Hahaha. I want to lose weight, I want body abs, ah, become fit.

Hanisah: that involves a lotof exercising, but okay. Thank you.

Transcript Respondent 10
Name: Siti Fatimah
Age: 23 years
Faculty/Program/Year: FSK/Biomed/4
Respondent Code: R10

Hanisah: Okay, I'm Hanisah. Can you please give me your name?

Siti: Siti fatimah

Hanisah: Do you exercise routinely? Meaning 2-3 times a week

Siti: 2-3 times? No. Sometimes

Hanisah: Okay. Um, sometimes? Usually, how many times in a week do you exercise?

Siti: Minimum 2. 2 times

Hanisah: But not this week?

Siti: Just once this week

Hanisah: Once this week. Okay okay. Define physical activities

Siti: Wow, this question is like… define physical activity? Activity that, uh, involves, uh, movement of body, uh, that cause some tension to the body. Something like that

Hanisah: Okay. Uh, define exercise

Siti: Exercise? Exercise is something that related to, uh, when you do a vigorous movement macam to…to do something like…exercise. Hahaha

Hanisah: Okay. What is the difference between physical activity and exercise?

Siti: Uh, exercise is more tense.

Hanisah: More intense?

Siti: Yes, intense.

Hanisah: okay, uh, why don't you exercise routinely? Or like what are the reasons that you feel a hindrance, why you don't exercise

Siti: Um, tiredness. Just finished class. That's it, the important reason. One more like… uh that's it. Tired after class. Because if I exercise in the morning, um…I'm lazy to wake up in the morning.

Hanisah: Okay. You feel like you do not have enough time?

Siti: Ah, yes. No time

Hanisah: Do, you have any other hindrance?

Siti: Um, I used to like going to the gymnasium but now because the equipment and the environment in the gym isn't good, I don't go anymore and also like, like, I am embarrassed. Embarrassed to exercise there. Because people know me. If it's at the outside gym, nobody knows me. Hahaha

Hanisah: Okay. Um, what are the things you do not like about exercise?

Siti: Don't like? Um, like what I said. I don't like people see me. One more, I have no friend to go exercising with. Hahahah. No gang and then if I exercise in my room, I also have no company so it's not fun.

Hanisah: Um, you feel how is exercise good for yourself?

Siti: Um, body…body shaping and um, health ah.

Hanisah: Okay. Apart from that?

Siti: Ah, apart from that, ah, stress reliever because we produce serotonin, um, happy hormones. Hahaha

Hanisah: Okay. What are the things that you feel would make you start exercise? That could motivate you to become more active.

Siti: When your body weight increases.. Hmmm, that motivates the most. And also when people say you have gained weight

Hanisah: Okay. So these things would make you exercise regularly? Any other things that would make you exercise regularly?

Siti: No, hahaha. Things like that, according to mood. Um, yes.

Hanisah: Okay. What kind of exercise do you like?

Siti: What kind of exercise, um, exercise that doesn't use, that doesn't involve running, that doesn't involve cardio. And I also do not like doing abs.

Hanisah: How about stretching, going up and down the stairs, you like?

Siti: Ah, that is okay. Um, the one that involve equipments. That is good too

Hanisah: You like those involving equipments?

Siti: Um, yes

Hanisah : Like rope jumping?

Siti: Yes, I like

Hanisah: Cycling?

Siti: I don’t like. Because it’s tiring. Haa Zumba is okay

Hanisah: Okay. Um, when we develop an exercise module, do you think you would be interested to join?

Siti: What kind of exercise module?

Hanisah: You imagine the exercise module is something like we give you a guideline, this week you will do this, then today, you will do like, and then you can do it on your own. And there will be days where we doo the activity in group. Are you interested? But it’s optional, whether to join or not. We just suggest.

Siti: I’m interested if there are things that we can do together. If on my own, it will depend on my mood to do or not. Sometimes I won’t do it

Hanisah: Okay

Siti: Need to motivate yourself, like high motivation to do it on your own. Hahaha

Hanisah: How do you feel you can motivate yourself?

Siti: For exercise? Um, like how? Maybe support from friends. Hahahaha. It’s like a support system, I think, when we exercise with friends, we have a gang. If I have friends, and if the place to exercise is satisfactory. Maybe we have interest to exercise. That would add to my interest to exercise.

Hanisah: Okay. If we ask you to exercise every other day, meaning you exercise, then you don’t, then you exercise, is it okay?

Siti: I’m okay with it

Hanisah: Um, this is considered a routine exercise, compared to what you are doing now. So what would motivate you to continue exercise that frequently

Siti: Push me, something like that. But like, after that like, uh, no really to motivate but something like..

Hanisah: Incentive?

Siti: Yes, like something to give us the will to exercise

Hanisah: What kind of incentive are you interested in?

Siti: Um, what? I don’t know. Um, don’t know. Hahaha

Hanisah: Okay, it’s okay. If in the exercise module we include running, like 5-minute running, is that okay?

Siti: 5 minutes is okay. Not more. Hahahaha. Because I don’t like running.

Hanisah: Okay.

Siti: Anything that involve cardio.

Hanisah: If it’s like endurance, you need to run with stamina, like that. Too long is not good?

Siti: Ah, too long not that good. It should be short and okay. Just steady.

Hanisah: How short?

Siti: Maximum half an hour

Hanisah: that is long.

Siti: Oh really? Ha okay. Maximum half an hour.

Hanisah: Okay. This is the last question, if you exercise what is your goal? Do you want to lose weight, get fit, build muscle, gain stamina or what?

Siti: Firstly because ah, exercise to release stress. Secondly, to reduce bosy weight, um, that it. That two.

Hanisah: Okay, thank you.

Transcript Respondent 11
Name: Syaira
Age: 23 years
Faculty/Program/Year: FGG/Pergigian/4
Respondent Code: R11

I: Assalamualaikum warahmatulallahiwabarokatu. I will be asking a few questions about activity, aaa, exercise. This session will be recorded. Do you agree to join?

S: I agree. I agree.

I: Okay. My first question, aaa, do you do exercise activities regularly?

S: Not quite regular, seldom.

I: Seldom. Okay. What do you understand about physical activities?

S: Physical activities are activities that gives off energy.

I: Energy. Okay. That's all. Okay. What do you understand about exercise activity?

S: Exercise activities? Exercise activities are aaa activities that requires energy but they are a bit leisurely. Meaning when we...

I: Like according to our suitability?

S: Yes. According to our suitability.

I: Okay, what. What is the difference between both?

S: Physical activity and exercise.

I: Yes.

S: Aaaa. For me, physical activities require energy while exercise require energy too, but less than physical activities.

I: Why don't you exercise?

S: Due to time constraint and laziness.

I: Time constraint due to a lot of assignments to do.

S:Yes. Because now when I got back from clinics, it's tiring and such.. so very seldom.

I: The laziness. Why lazi?

S: Why lazy? Sometimes after class and tired of studying, I feel lazy to go out. These days I rather sit down in the room rather than walk and do exercise

I: in summary, because of tiredness

S: Yes. Because of tiredness

I: What don't you like about exercise?

S: Hmmm. What don't I like, but I like exercise actually

I: Maybe because it requires place or because of the sweating and tiredness?

S: No. Not really

I: No

S: Maybe exercising in public that I don't like

I: Basically, like an open space.

S: Yes. I don't quite like open spaces

I: What. In your opinion, what does exercise give you? Like its benefits

S: Aaa. Feeling healthy, sweating, active. Feels happy. I feel that my body is healthy. Do you understand? Feels more energetic

I: Feels like our body is healthy, gives energy

S: We feel like our legs and hands are stretched, feels like.. do you understand?

I: They become more active, and we use that

S: Haah. Yes. Correct

I: What drive you to start exercising?

S: Aaa. When I have free time and feels like exercising. And because I haven't exercise for a while, not sweating for a while

I: Like.. Maybe there's another reason? Like feeling healthy?

S: Yes. The main reason is to be healthy

I: Or to lose weight?

S: No. No, because to be healthy

I: Or because you friends invite you?

S: No. Because to be healthy, probably

I: If you start exercising, what would make you want to do it again? Keep doing it

S: Because I can feel like. Feel like healthy. Feels sweaty

I: Sweaty

S: Feels. Feel more energetic

I: So aaa umm it's the effect of the exercise

S: Effect of the exercise

I: Effect of energeized

S: Haa. When we study at night, we don't feel tired, feels the eyes like aaa, we don't feel tired to study. Can you understand me? Feel energized, the brain is smarter

I: Okay, is that all?

S: Yes. Haa

I: What kind of exercise do you like?

S: Aaaa

I: For example, climbing..

S: Play. Exercise is not play?

I: Play is included

S: Play anything that makes you sweat. I like playing futsal, like it. I don’t run much

I: How about sit ups?

S: Sit ups is fine. Sit ups. Rope skipping

I: Rope skipping. How about climbing up and down the stairs?

S: Sure. Treadmill

I: How abouit jogging, do you like?

S: Haa. Don’t like

I: Okay. If I produce an exercise, exercise program. What would make you want to join the exercise program?

S: If the exercise is like zumba

I: Zumba. With dance moves. With music?

S. Haa. But only for females.

I: Only females. Anything else? Maybe due to movements?

S: Is yoga exercise?

I: Haa. Yoga is an exercise

S: Haa. Yoga. I’ve always wanted to try it

I: If it, that thing, the exercise is interesting, attractive, like easy to do?

S: Attractive. Easy to do?

I: like if it’s easy to do on your own or it can be done in the room, like that?

S: Aaaa. What kind of exercise?

I: Not exercise. What would make you to join the exercise?

S: Aaa. Because of exercise that. That draw sweats

I: So it doesn’t matter if it’s easy or difficult?

S: Aaa. Not too difficult. Things we can do

I: That we can do

S: Not too much

I: Not too much. Can be done

S: Haa. That can be done

I: How about the place. Like you need a larger space of just a small space?

S: Large space. Better

I: Anything that you want to add?

S: Nothing else

I: Okay. If the module requires you to exercise today, rest tomorrow then you exercise again. Would you do it?

S: Aaa. Yes

I: Yes. How long do you think you want to do it?

S: Do the exercise?

I: Yes

S: About an hour

I: Okay. What would you. What would make you follow the exercise always?

S: If it's interesting. Aaa. If the exercise is done at a suitable time, meaning I can join. Aaa like easy for us to do at a suitable place, it's okay. I will join

I: Basically, suitability of place and time?

S: Yes

I: Okay, that's all the questions. Thank you.

S: You're welcome.

Transcript Respondent 12
Name: Chan
Age: 23 years
Faculty/Program/Year: FSK/Biomed/4
Respondent Code: R12

Hanisah: Hello, this is Hanisah. Can you please give me your name?

Chan: Ah, Chan Chin Yi.

Hanisah: Can you define physical activity?

Chan: Physical activity is, uh, activity that like you stretch your body or running or jogging then, ah, can increase your metabolisme and then at the same time you sweating also you can ah, detoxify.

Hanisah: Ah, what is exercise?

Chan: Exercise…exercise is like for example, jogging, ah, do some like, yoga, ah, exercise can be divide into like heavy exercise or light exercise.

Hanisah: Ah, is exercise the same thing as physical activity? For you lah.

Chan: Um, for me it's a bit different. Ah, exercise is…physical activity for example, you walking, you brisk walk like normal walking, but exercise is like, uh, require some like exercise is more…require like, the strength is different I think.

Hanisah: Intensity?

Chan: Ah, intensity is different.

Hanisah: Okay. Do you exercise regularly? Like for example, um, this past week lah, this past 7 days, how many times in a week have you exercised?

Chan: No.

Hanisah: Okay lah. So you say you don't exercise regularly lah?

Chan: Yes.

Hanisah: Okay. What are the reasons that you don't exercise regularly?

Chan: I think, first is the self-discipline because like I don't have the discipline to motivate me to like exercise every day. And then time-constraint, because of like, uh, whenever like get back from college, then will feel tired right? And then I will rather choose sleeping than go and exercise.

Hanisah: It's okay. What are the things that you don't like about exercise?

Chan: Don't like ah? Don't like?

Hanisah: Like you said just now it's tiring…

Chan: Ah, maybe ah, don't like, it's like, after exercise you feel muscle pain, uh, like that. That's the part I don't like.

Hanisah: Okay. How do you think that exercise is good for you yourself?

Chan: Ah, as I said because exercise can help us to increase our metabolisme, and then at the same time, if it can like, help us to prevent some disease like if we have high…high fats content, we can burn…burn, not burn calorie like, when we sweating we can detoxify after that, we can easily to get fit.

Hanisah: Ah, do you like exercise?

Chan: Ya, I like exercise but like, I don't have discipline to motivate myself.

Hanisah: It's okay. Um, what can you do to start exercising? To motivate yourself to exercise.

Chan: Uh, motivate myself…Maybe like, uh, make it like a habit like uh, do it for, uh, because I habits is build up within 21 days right? Maybe I continuously do like day by day then become a habits then it become naturally, exercise is like natural things for me.

Hanisah: Um, what kind of exercise do you like?

Chan: I love like uh, open a video then dance together like call what ah that one? Aerobic, ah, is it aerobic? I don't like, like jogging, eh not jogging. I don't like running because run very tiring. I love, like dance more. Aerobic lah. Aerobic kind.

Hanisah: Um, what about stretching, or something like yoga, do you like that?

Chan: Ya. Also okay.

Hanisah: Um, what about uh zumba?

Chan: Zumba like fast, like dancing right? Okay. Prefer that type.

Hanisah: What about going up and down the stairs?

Chan: Going up and down the stairs also okay. Running I not that, not that love running. Like running lah. Marathon. That one is more…intensity is like high higher ah.

Hanisah: Do you prefer exercise alone in your own room, in your own comfort zone or exercise like as an activity in a group?

Chan: Uh, If I had a friend who had similar interests with me, I would rather exercise in a group because we can give each other motivation because we are friends.

Hanisah: If we make an exercise module and we ask you to exercise every 2 days, meaning you exercise then you don't exercise, then you exercise again. Would you join?

Chan: Ya, most probably because like it's a program like then you need to committed to the program. So, like it's kind of like discipline yourself only. Like like force like the push, move, like the force lah, got something trigger you to exercise, to go exercise.

Hanisah: Okay, so the program is like your motivation?

Chan: Yes.

Hanisah: Um, should we include running in the exercise module that we make?

Chan: Uh, running ah? Can but not that, not the distance that is very far, or can slow running rather than like run fast.

Hanisah: How much time would you spend on running?

Chan: Running ah? 30 minutes maybe. Slow running. Like slow jog.

Hanisah: Okay. Um, the last question is what is your goal when you exercise? Do you want to increase your mobility, or increase your stamina, or do you want to build muscle, or do you want to lose weight, or do you want to just get fit?

Chan: Get fit.

Hanisah: Okay, thank you for your time.

Transcript Respondent 13
Name: Amalina
Age: 23 years
Faculty/Program/Year: FSK/Opto/4
Respondent Code: R13

Interviewer: I'm going to ask a few questions about physical activity. Okay. This interview session will be recorded. Do you agree?

Respondent: Yes.

I: Do you exercise routinely?

S: No.

I: No. Okay What do you understand about physical activity?

S: What I understand about physical activity...Physical activity is an activity that we do in an organised manner. This means that we plan the activity and do it in a routine manner. I mean, maybe 3 days in a week, done in a routine way, not like today you want to do it but tomorrow you don't.

I: What about exercise? What do you understand about exercise?

S: The exercise that I understand is a body movement that we plan, I mean the movement is organised.

I: So what is the difference between the two, physical activity and exercise?

S: The difference between physical activity and exercise...physical activity is more to the movements that we do, daily activities like walking is a physical activity but exercise are movements that are, are vigorous. Well maybe not vigorous lah but movements that are different from our daily activities, like that. That we planned.

I: Why don't you exercise?

S: Why don't I exercise...For now, it is the rainy season. Secondly, I don't exercise because I'm lazy.

I: Laziness. Could you say that it is because you don't have enough time?

S: Not really. Because now we are on holiday so there is time but because there is so much time, I'm lazy. Yeah, lazy.

I: Lazy. there aren't any other reasons?

S: No. Time restraints only happen sometimes but I mostly only do activities in my own room like zumba in my room but not in a regular manner. I don't have a fixed routine or anything. If I feel like doing it, then I do. If I don't feel like doing it, then I don't, like that.

I: What are the things that you dislike about exercise?

S: What I don't like about exercise. What I don't like about exercise..What I don't like about exercise. Exercise ha...exercise makes me tired but I like sweating.

I: Is there any other reason?

S: No.

I: Just because of tiredness.

S: Haa. Because, I don't know why.

I: Or because after exercise, our body feels sore? Or we feel like we don’t have energy?

S: Aaa. No. Because exercise makes me tired but I like sweating. That is all.

I: That’s it. Okay. What do you feel that exercise, uh, what are the benefits of exercise for you?

S: Usually exercise will make me less stressed and I’ll feel more energised after exercising. Then, exercise makes me feel relaxed after that.

I: Is there anything else?

S: What I mean is, it mostly makes me feel relaxed after sweating. It definitely makes me feel relaxed. That’s all.

I: That’s all. Okay. If you exercise, what do you think are the reasons that you begin to exercise?

S: What makes me exercise. When I feel that I am physically weak. When I feel unhealthy I will start to exercise. What I mean is when my body feels like it is feverish maybe, or I have a headache maybe, times like that.

I: Are there other reasons?

S: There are. Sometimes, I do it because I want to be slimmer but if it is for that reason, it won’t last that long.

I: Is that all? Are there any other reasons?

S: No.

I: What helps you to continue exercising? Like to make exercise a regular thing.

S: If. If there is anything, what do people call it, the thing to run on.

I: Treadmill?

S: Haa. Yes. If there was a treadmill. If there was a treadmill in the house or in the room, then it would be easy, right? I wouldn’t need to leave the house, it would be convenient for me.

I: That’s all?.

S: That’s all.

I: There’s nothing else? Maybe reasons like because of friends or...

S: No.

I: No. Or because, ohhh, to maintain or to see the results of exercise, something like that?

S: No.

I: Okay. If I want to produce an exercise module, what would make you want to join the exercise module?

S: What would make me want to join the module. What would cause that. This question is very hard. Give me a minute to think.

I: Okay. Sure.

S: What would make me want to join the exercise module. Maybe if the exercise module is done in a group, maybe. What I mean is that, not just giving me the module and expecting me to do it. I mean, It would be easier if there was someone to teach me to exercise and what to do. If I'm alone, it is much harder to learn how to exercise.

I: Other than that?

S: Other than that, the exercise module must be easy to follow and understand, meaning that it isn't burdensome. I mean, that it doesn't require all those weird equipment and all that.

I: What about a place to exercise? Do you need a wide area maybe or can you exercise in your room?

S: I can. Okaylah, I can do it in my room but like I said just now, I prefer if the exercise module is not exercising alone.

I: Like there should be a friend who does it with you.

S: Yes. Meaning that we should do it in a group lah so we don't have the same target. Meaning that, it would be fun to have friends together with you.

I: If the exercise module required you to exercise on alternating days, meaning that, you exercise today, rest tomorrow, then exercise again the day after. Would you do it?

S: It depends on my time restraints. I mean, if I have the time, then I can exercise, maybe, maybe I'll exercise.

I: Depends on you time, then?

S: Yes, depends on my time.

I: Say, for example, we set a cumulative amount of time in minutes that you have to exercise in a week, and then you could do it during your own free time. Would you do it?

S: It can be considered. Maybe I can do it.

I: What would keep you motivated to continue exercising with the exercise module?

S: What, what would cause it. Maybe. What ah, maybe I'd have to set a goal. I mean, to do the exercise, what is the goal. A goal that seems possible to achieve, something that we can see. Maybe say, we know that weight loss is the goal is the goal here now, we, what is the goal after that. Then, another one would be, there has to be someone who can frequently remind us of that. Like, ohh, exercise. Did you exercise yet today? Like always reminding. Sometimes, it's not that we forgot, we remember to exercise but it's that we conveniently pretend that we forgot to exercise.

I: Is that all of it? Is there anything else that you would like to add?

S: Maybe there has to be an external force.

I: Okay. Thank you. That is all.

Transcript Respondent 14
Name: Tan Jiah Ning
Age: 23 years
Faculty/Program/Year: FSK/Biomed/4
Respondent Code: R14

Interviewer: Okay, this is Hanisah. Please give me your name?

Respondent: I'm Jiah Ning.

I: Okay, Jiah Ning, do you exercise regularly like 2-3 times a week?

S: Yes.

I: This week how many times did you exercise?

S: Uh, this week? For this week it's basically it's no, but uh, previously during the weekday I will go for taekwando training for 3 times a week.

I: Um, okay. Define physical activity.

S: Physical activity…hahaha…is something that we move our body to do the work, uh, can be light, or heavy, ya…okay.

I: Ah, alright. Don't worry there's no right or wrong answer. Ah, define exercise.

S: For me exercise is something that will make us feel, uh, a bit refreshing and increase our heartbeat, or the breathing rate and make us sweating, um, ya.

I: Okay, what's the difference betwenn physical activity and exercise?

S: Hahaha, for me, physical activity is like, um, all the movement, physical activity is all like, the movement we do in our daily life lah. And then the exercise is something that we do in extra to, uh, to…

I: To be fit?

S: Fin?

I: Fit.

S: Oh, fit, ya. It's for fit, for increase the fitness or for sweating lah.

I: Okay, that's good. What makes you want to exercise routinely?

S: Um, to keep fit. And actually while exercising is uh, it's fun for me lah. And while sweating it makes me feel more refreshing.

I: How many times do you usually exercise in a week? Despite the last week lah.

S: 3-4 times.

I: It’s quite frequent.

S: um, do you feel you exercise enough?

I: uh, hahaha, if I go training everytime, I feel it’s enough. But sometimes maybe I will skip the training.

I: So, ideally for you is 3-4 times. What motivates you to exercise continuously like not skip training?

S: Um, for me, exercise is like a discipline, we have discipline to keep moving us, to do the exercise regularly. So, and the discipline can be applied in all the other stuff, in our daily life, so I feel like this is my responsibility to do the exercise.

I: Okay. Ah, what are the things that make you stop exercising or it makes you feel like you want to skip?

S: Laziness? Hahaha.

I: What makes you feel lazy?

S: What makes me feel lazy ah? Uh, if I keep exercise for 1 period continuously I will feel like I lose the uh, energy to do it continuously. And also if the, like the, uh, academic work is too heavy or activity or co-curricular is too many then I will skip, I may skip the exercise.

I: Do you feel like you don’t have enough time to exercise?

S: Don’t have enough time ah? Actually, no because for me exercise can be uh, short or long right? So it’s just the matter you want to take the initiative or not.

I: Okay. Very interesting words. Um, in your opinion how do you think exercise is good for yourself, like how do you benefit?

S: Sorry?

I: In your opinion, how do you think exercise is good for you? Like how do you benefit from the exercise that you do?

S: Um, the main benefit I think is can keep fit, and then like uh can make my mind more clear and concentrate. And also I can feel, uh, happiness when I exercise. As you know we will have that endorphine while exercise right? And then, and also, while exercise I can make friends. To be with my friends and uh, I think it is a good time when exercise.

I: Do you prefer to exercise like by yourself like in your own room, in your own comfort zone or do you prefer to exercise as an activity in a group?

S: If individual exercise, I prefer to be outdoor like jogging in the park, instead of in my own room. Got for the group exercise I also like, yeah, and actually I prefer group exercise.

I: Um, if we do, we're gonna do an exercise module for female students who live in a college, if the exercise module needs you to exercise every 2 days, meaning you exercise, and then you rest, then you exercise. So, it's quite frequent. Do you want to do it? And why?

S: Uh, I cannot promise. Hahaha.

I: Because it's time consuming?

S: Yeah. And I can't sure for next semester I can, uh, cooperate or committed for this exercise module. But if the time is available, maybe I can, I can join.

I: If the exercise is something where we give you a guideline of how to exercise, we teach you how, then we just let you exercise by yourself. Do you think you would want that?

S: Uh, hahaha, maybe.

I: Okay, I'll take a maybe. It's okay. What kind of exercise do you like?

S: Uh, the exercise that is vigorous maybe.

I: For example?

S: For example ah? Jogging…jogging is vigorous or not?

I: It depends on the speed and the time and how much you jog but usually its moderate.

S: Um, ya, then, almost moderate. Moderate exercise.

I: What about stretching, zumba?

S: Stretching is okay but I never try zumba. Hahaha

I: Are you willing to give it a try?

S: Ah…..(seems like no from body language)

I: Okay. Um, what about going up and down the stairs, jump rope?

S: Can.

I: Okay. Ah, what do you dislike about exercise?

S: Dislike about exercise? Nothing. In terms of what?

I: Um, like are there things that you don't like about exercise like you feel pain afterwards or you don't like sweating or stuff like that.

S: Basically no. but, uh, if got some of the most I don't like is uh the injury. Hahaha

I: Oh. Ya, injuries suck. Aside exercise, what other physical activities do you like?

S: Aside exercise? What physical activity I like? Uh…hahaha what is the question again?

I: Um, not sure?

S: Ya, not sure. Sorry.

I: Ya, okay. It’s okay. Thank you for your time.

Transcript Respondent 15
Name: Kristina
Age: 23 years
Faculty/Program/Year: FGG/Pergigian/2
Respondent Code: R15

I: I will be asking you a few question about exercise. This interview will be recorded. Do you agree?

S: Okay.

I: What do you understand about physical activity?

S: Physical activity where you strain yourself to do something for your physical fitness.

I: What do you understand about exercise?

S: Exercise is something related to physical fitness. It kinda like physical activity.

I: So how do you differentiate between them those two?

S: exercise is just, how to say. You just wanna sweat like that. It main purpose is not to get physically fit but physical activity you will do,you those things to get fit physically.

I: Why do you. Do you exercise often? Routinely.

S: Not really because my schedule is quite busy but I try to exercise once in a week.

I: Why do you usually not exercise?

S: Because exercise release stress, some more if you stuck in your room for so long you will feel stress facing the four walls. If you go out and enjoy the view, exercise is kind like positive vibe.

I: What stops you from doing exercise?

S: It’s like timing. Class timing.

I: Class timing.

S: My usually 8 to 4 somtimes it will take 5 to reach my room. So after that i will be like so tired to do rountine like daily schedule. I will just sleep.

I: Basically no energy. How about maybe because of places?

S: Places. Yea inside got field. Inside our collage got field but it not well maintained because I prefer to go out to the tasik. Instead of exercising here. Because I enjoy the view at tasik.

I: What you dont like about exercise?

S: Not really.

I: Not really. Do you not anything? Becuase you feel tired after exercising or sweating?

S: Tak.

I: No. What is the benefit of exercise for you? Opinion.

S: We will not be easily tired although we have class 8 to 5, 8-4. Not easy to be tired. Still got energy. Can push.

I: That is it?

S: Hmm.

I: That is it.

S: Ya.

I: Nothing to add on?

S: Nope.

I: What would you make you to exercise? Start exercising.

S: Start exercising. Becuase it is like my inner instinct. If tak sweat for so long toxin will trap in our body. Once you like sweat, you can feel it like refreshing.

I: How about goals for you starting exercising? Goals to carry out exercise.

S: Actually my goal is to be physically fits lah.

I: If dah start exercising, what can help to maintain the exercise?

S: Self motivation.

I: Other than that maybe. Because of the routin of exercising?

S: Not really.

I: Self motivation only. What is your favourite type of exercise?

S: Normally i do jogging. And then just like workout.

I: Up and down stairs?

S: I dont do up and down stairs. I dont allocate time for it but when in faculty and college i dont take much lift lah. Try.

I: If like skippig rope?

S: Skipping rope. Dont like.

I: Stretching?

S: Before. Stretching sometimes lah.

I: If I do one exercise module, what makes you want to follow the exercise module? What will make you follow the exercise module tu?

S: Determination.

I: Determination?

S: It’s like okey we should, how to say, constanly do the thing. Another one is motivation. It because it for our own good right.

I: If from the aspect of the exercise module, maybe its context is more interesting or easy?

S: Not really.

I: Not really. Mostly motivation lah. If the module requires you, today exercise, next day you take a rest and then you exercise again. Will you follow it?

S: Yeah.

I: How much time will you allocated in for the exercise?

S: If per day 30 minitlah maybe. So we are students like and we got lots of other things to do.

I: What motivate you to exercise, like exercise today, after that next day rest and continue exercise?

S: Because muscle need time to rest. So I will just. Because after exercise you can feel the pain sometimes if beginners lah. You can feel the pain so like for the muscle to recover we need to rest.

I: If the module if self is intersting ka? Macam kalau dia suka buat benda tu ka, suka exercise ka, it’s fun ka macam tu?

S: Yea, it is fun I will do lah

I: Is there anything you would like to add?

S: Not really.

I: Okay. Thank you very much.

Transcript Respondent 16
Name: Hui Yee
Age: 23 years
Faculty/Program/Year: FSK/Biomed/4
Respondent Code: R16

I: Hai. I am Ida. There are few questions on exercise activities. This session will be recorded. Are you okay with it?

S: Yeah. I'm okay.

I: Do you exercise regularly?

S: Not really.

I: What do you understand about physical activity?

S: Physical activity is the activities like we actually sweat, release sweat and then with the aim to have a fit body or healthy body.

I: What do you understand about exercise?

S: Exercise. Exercise. Exercise almost the same with physical activity and then exercise there can be very vigerous or like the calming one like yoga or calm like..or vigorous.

I: So in your opinion what's the difference between these two?

S: Different between the physical activity and the exercise. I think for physical activity, would be, it might be more to, more to the vigorous one lah and then exercise it can be both. Well exercise is more general, more widescope.

I:Why dont exercise?

S: One of the reason maybe lazy.

I: Lazy.

S: Laziness. This actually the major problem.

I: The other problem?

S: The other problem maybe I don't take inisiatif like even though we know, I know exercise is very important but I didn't initiate it.

I: How about time constraint?

S: Sorry?

I: Time constraint. Is time constraints becoming one of the reasons?

S: I don't think myself take it as a very important thing. So I don't spend masa for it.

I: What you don’t like about exercise?

S: Sorry.

I: What you don’t like about exercise? Maybe because of swaet ka, tired ka, pain ka?

S: Swaet kut. Maybe don’t like but how can I say that?

I: Is it tiring ka? Like that.

S: No.

I: Ka neutral?

S: Neutral.

I: In your opinion, what, what exercise can be good for you? Like benefits of exercise to you.

S: The benefits of exercise can help maintain our body condition. I mean in a very good physical with a good physical appearance and also to make us to maintain a fresh mind.

I: What will make you start exercising?

S: Bila I dah rasa, our, my condition, maybe the weight has been. Maybe.

I: Motivation lah?

S: Maybe lack of some motivation.

I: How about friends? Friend to help you exercise? Friends

S: Maybe I don’t focus on that side maybe not. Maybe peer pressure.

I: How about goals? Like reducing body weight?

S: Yea. There is a goal always but no motivation.

I: The main reason to start exercise. what is the biggest cause?

S: Yang trigger saya exercise. Myself awareness.

I: Self-awareness. When you start exercising, what would keep you exercising?

S: When I make this thing very important and it became a part of my life. Now maybe because saya choose for not facing to reality.

I: Other than that maybe facilities or there a coach? Like got personal coach helping you. Things like that.

S: I think maybe got personal coach is better for me?

I: Ha. Yea?

S: To like motivate me?

I: Haa. Ya.

S: That one of the choice, one of the method also lah for me to aware about.

I: What type of exercise to you like?

S: Zumba.

I: Zumba. Okay.

S: I actually do Zumba at home town but at here no. Because at home will follow my mum go Zumba. At here no motivation.

I: Macam jogging?

S: Jogging?

I: Suka tak?

S: Not really lah. Like dancing like that.

I: Up and down stairs? No? If skipping?

S: Skipping?

I: Jump rope? Sit Up?

S: Sit Up. Okay.

I: Stretching?

S: Stretching okay.

I: Push Up?

S: Push up. Not really.

I: Not really. If we produce one module right, and what will make you follow the module?

S: The time is flexible. Mean module we can do the module whenever we want. That the main point lah.

I: The other point?

S: The exercise I prefer.

I: If exercise too easy to follow and you can do in your own time, if you can do in your own room?

S: Yea.

I: If the exercise is interesting like that.

S: Maybe like special activity which I never done before. Mean like have more adventurous.

I: If the exercise module requires you, today you exercise, next day you rest then following day you exercise will you follow it?

S: Yea. It better.

I: How much time will you willing to spend to exercise?

S: Mungkin ½ hour.

I: what keep you exercising, after if the module like today exercise, tomorrow rest then exercise? Like normally people after one-day exercise, the next day will rest. The third day want to exercise back will feel like lazy. What causes that? Also wanted to know what motivates to continue exercise although resting for one day and supposedly continue back the next day?

S: Because today already rest so tomorrow I think still can follow the module.

I: If from the aspect of the module? Like maybe from friend's aspect? or your self-motivation, or with any goals?

S: Because if I choose to follow the module I will follow what's in the module.

I: Okay that's all. Thank you.

Transcript Respondent 17
Name: Hafiqah
Age: 22 years
Faculty/Program/Year: FSK/Biomed/4
Respondent Code: R17

I: Bismillahhirrahmannirrahim. I will be asking a few questions about exercise. This session will be recorded. Do you agree to join this session?

S: Yes

I: Hafiqah right?

S: Yes I am.

I: Do hafiqah carry out exercise activity routinely or not?

S: Exercise activity routinely. Done that before. Indeed, doing that daily in the bathroom. I does stretching. Exercise in the bathroom.

I: What does Hafiqah understand about physical activity?

S: Physical activity, activity which uses physical.

I: What does Hafiqah understand about exercise?

S: Exercise. Exercise is a way to maintain healthy. Exercise is an activity to maintain healthy.

I: To make us healthy. So what is the difference between physical activity and exercise? Based on Hafiqah's opinion.

S: In my opinon, exercise is, don't know.

I: Don't know. Ohh its okay. When Hafiqah starts to do exercise? When? Meaning how long already?

S: Leaving or doing?

I: Doing?

S: Just now in the morning.

I: No, when you start doing exercise.

S: Oh. When starts doing. Should be earlier this month.

I: why Hafiqah starts doing exercise?

S: My body weight increases.

I: Body weight increases. That's the only reason.

S: Because that day with beer belly. Wanted to maintain body shape.

I: How many times Hafiqah does the exercise?

S: I does it every time I bath. I bath 2 times per day, so 2 times per day.

I: In a week?

S: 2 time 7. 14

I: 14. Okay. Does Hafiqah feels that the exercise is enough?

S: No.

I: No. Why?

S: Because my beer belly is not missing.

I: What helps Hafiqah to maintain exercising?

S: Desires to reduce belly.

I: Desires to reduce belly. Okay. Apart from that?

S: Apart from that, to maintain healthy.

I: Maintain healthy. Okay. Any reasons to reduce body weight?

S: Just wanted to reduce body weight.

I: Or self-conscious?

S: I felt I am not conscious.

I: Or maybe friends?

S: Friends. No.

I: How about the place?

S: Place. Ya place. I have roommate so if wanted to exercise in the room I feel shy.

I: Okay. What stops Hafiqah to always exercise?

S: Because of place. No place to exercise. And second thing is lazy.

I: Second thing lazy. Any other thing? Like need equipments?

S: No facilities to exercise.

I: Or feel need money for exercising?

S: No. Exercise dont need money.

I: Okay. In Hafiqah's opinion, what is the benefits of exercise to Hafiqah?

S: From stamina aspect.

I: Stamina?

S: If I exercise, I will get like in a day, I can maintain my stamina. For example, do labwork. Will not easily feel tired. And secondly, maybe if I exercise, will catch less sickness.

I: Sick. Anymore?

S: That is it.

I: That is it. No like wanted to maintain fitness?

S: That is it. One of it is also to maintain fitness.

I: Or want to maintain

S: Body shape?

I: Ha. Body shape.

S: Haa. Maintain body shape.

I: If we do one exercise module which requires Hafiqah to exercise interval days. Is Hafiqah willing to do so?

S: The exercise module is light or heavy?

I: Are you wiliing to do?

S: Interval days?

I: Haah

S: Interval days?

I: Haah

S: In one week, interval days.

I: Haah. Not willing to do? Why?

S: Because of time unless if the exercise only needs few minutes then I can do.

I: Meaning about how many minutes?

S: About 15 minutes. 15 to 20 minutes.

I: 15 minutes. Okay. How about the exercise? Any needs for like brief or interesting activities.

S: The exercise do not need any equipment. Only with ourselves.

I: Only ourselves. Need music together?

S: No. Don’t mind. I don’t mind.

I: Don’t mind. So the exercise needs…

S; But if there is music I feel that is more okay. It can like help us to feel fun exercising.

I: What makes Hafiqah maintain exercise at interval days.

S: Maintain healthy.

I: Maintain healthy. More for example got friends to exercise with us or no? Or Hafiqah’s opinion is preferring exercising alone?

S: Alone. I likes to exercise alone. That’s why I exercise in bath room/

I: if exercise location like…

S: Closed area.

I: Closed area. Like need little space?

S: Haa.

I: Need little space.

S: Since I like to exercise on my own I like closed places and places that are big enough for me.

I: Small space.

S: Haa. Small space.

I: What types of exercise Hafiqah likes?

S: Exercise. Need to talk about specific types of exercise activities or how?

I: Can just state.

S: Type of exercise. What is the name? I do not know the name but only know the movement. The movement that touches the tip of the foot.

I: Like stretching.

S: Haa. Like stretching.

I: Stretching. If like up down stairs?

S: Don’t like.

I: Don’t like. Aaa, sit up?

S: Like.

I: Like. Zumba?

S: Don’t like.

I: Don’t like. Yoga?

S: Yoga likes.

I: Like. If pumping?

S: If pumping.. likes.

I: if cycling

S: Cyling likes. Like that. Walking.

I: Walking.

S: Walking haa.

I: Okay. What things that Hafiqah do not like about exercise?

S: Don’t like about exercise. Easy to be tired. Just do exercise for 5 minutes like that if it is vigorous. Get tired quickly.

I: What about pain?

S: Pain. If stretching there will be no pain. But if jogging there will be some pain.

I: What about sweating?

S: I don’t mind about sweating.

I: Don’t mind.

S: I like to sweat.

I: What is Hafiqah’s favourite activity apart from exercise?

S: Watching movie.

I: Watching movie.

S: Sit and daydreaming.

I: Like gardening?

S: I like gardening but I do not have the chance to do gardening.

I: If walking and looking at the sceneries?

S: I like walking without destination. Don’t like watching sceneries. I like to walk in the morning only, not afternoon.

I: Anything to add on?

S: What else? The activities that I like?

I: Haah.

S: Washing clothes.

I: Washing clothes.

S: Using hand.

I: Okay.

S: Drying clothes.

I: Like tidying room?

S: I like to tidy room.

I: Just like that. Thank you.

S: Welcome.

Transcript Respondent 18
Name: Saadah
Age: 23 years
Faculty/Program/Year: FGG/Pergigian/3
Respondent Code: R18

Hanisah: Okay, this is Hanisah. If this interview is recorded is that okay?

Saadah: Can.

Hanisah: Ah, Can I have your name?

Saadah: Ah, Wan Nur Saadah binti Wan Zamri. FGG/3.

Hanisah: Okay, um, for you what is physical activity?

Saadah: Physical activity. Like exercise. Exercise can release the sweat. Something like movement can help sweating.

Hanisah: Okay. What is exercise.

Saadah: eh, just now is physical activity right?

Hanisah: haah.

Saadah: Exercise? Exercise… ah, about the same also. But it is not spontaneous. Like we need to train. Need to plan. Ha. Need planning.

Hanisah: Okay, ah, do you exercise routinely?

Saadah: Ah, not maintaining often but about 3 times a week. Mostly on weekends.

Hanisah: Ah, is it like you will exercise about 2-3 times a week and each time at least 30 minutes?

Saadah: No. 5 minutes a day?

Hanisah: Okaylah. At least got. Uh, why don't you exercise um, longer? Any reasons?

Saadah: Because of one thing only. Because lazy. Hahahaha. If watch video…exercise videos, body *unclear* that type, will be motivated. After that only for a while motivated. Then discontinue. If do also for a brief while because of lazy. Just lazy.

Hanisah: Okay, um, what you don't like about exercise?

Saadah: Um, if got people because I am abit shy.

Hanisah: Um, why shy?

Saadah: Because…because why? Not sure about why shy. Because the other people is not exercising also. So if I wanted to do exercise I become shy because others are not doing so.

Hanisah: Um, do you feel shy about your body shape?

Saadah: Not really. Just abit.

Hanisah: Okay, um, how do you think exercise is good for yourself?

Saadah: Um, um how ah?

Hanisah: What can you get if you exercise?

Saadah: What can I get eh? Get get get get… Can reduce body fat if jogging. But I rarely do so. But it is in my plan.

Hanisah: Can you relieve stress if exercising?

Saadah: Relieve ah, sometimes.

Hanisah: Okay, ah, what else that you can do to motivate yourself to exercise?

Saadah: Um, look at pictures of fit people. Hahahaha… There are six packs, ah, that's it.

Hanisah: Okay, ah, apart from that, what others can do so that you are more motivated untuk exercise?

Saadah: Others also exercise, so like inviting to exercise together. Ha, can also.

Hanisah: You prefer exercise alone or as a group activity?

Saadah: Actually group activity but always alone only. Because there is no other people exercising.

Hanisah: Okay. Um, what kind of exercise do you like?

Saadah: Um, plank, push up, actually I am not sure what is the name if the exercise I am doing.

Hanisah: Ah, it’s okay. Um, what about up and down the stairs, jump rope, stretching, uh, running?

Saadah: When it comes to going up and down the stairs, confirm I feel so lazy. If there was a lift, I would definitely take the lift. I can’t bear to do high intensity exercise. But I will do stretching. Ah stretching.

Hanisah: Ah, jump rope?

Saadah: No.

Hanisah: Running?

Saadah: ah running. Not very.

Hanisah: Um, zumba?

Saadah: Ah, likes zumba.

Hanisah: Okay, um, next sem we will do one exercise module for female who stays in KTSN. Do you want to join? And then, what you want in this exercise module?

Saadah: Want to join but depends on time, ah, if got time, ha, can join. And what I want eh? a lot of people… ah groups lah.

Hanisah: Would you exercise every 2 days? Meaning that if exercise, rest, exercise, rest. Will it be too frequent?

Saadah: Ah, that is too frequent. Normally only weekends.

Hanisah: Um, okay. Should we include running in the exercise module?

Saadah: No. Hahahaha.

Hanisah: Okay. Last question. What is your goal when exercising? To lose weight, or get fit, ah, stamina? Build muscle? What is it?

Saadah: Untuk shaping kot. Ah, muscle lah.

Hanisah: Ah, okay, thank you.

Transcript Respondent 19
Name: Amirah
Age: 22 years
Faculty/Program/Year: FSK/EVH/4
Respondent Code: R19

Hanisah: Okay. Can this interview be recorded?

Amirah: Can

Hanisah: Okay. this is Hanisah. Can you please give me your name?

Amirah: My name is Anith Amirah.

Hanisah: Okay. Please define physical activity.

Amirah: Ah..Okay. In my opinion, okay. Physical activity, is the activity which normally people do to make our body fit, to maintain our body weight.

Hanisah: Okay, please define exercise.

Amirah: Exercise… Ah, okay. Exercise is like meaning…Ah okay. Exercise…ah, cannot lah. Okay, normal movement which people normally does like jogging, running, cycling like that.

Hanisah: In your opinion, what is the difference between physical activity and exercise? or for you it is the same?

Amirah: Um, feels like the same.

Hanisah: Um, do you always exercise routinely? Which is 2-3 times a week.

Amirah: Um, no.

Hanisah: Okay, what is the reasons that you do not exercise routinely?

Amirah: Busy, um, sometimes lazy. No time.

Hanisah: Okay, um, what is that you do not like about exercise?

Amirah: Haha. Tiring. Sweating ha.

Hanisah: Okay, um, for you, how is exercise good for yourself? What else that benefitted you if you exercise?

Amirah: Okay if exercise normally its benefits are like, um, can make our body fit, maintain our body weight. Um…hahaha

Hanisah: Okay.

Amirah: Feels like that’s it. Haha.

Hanisah: Um, what else that you can do to motivate yourself to start exercise?

Amirah: Um, for motivation, if someone invites me to exercise then I can follow them but if no one invited me to exercise, I will definitely not exercise by myself.

Hanisah: Okay, um, besides from friend's invitation, got any other reason which can makes you exercise routinely?

Amirah: No. Really no.

Hanisah: Um, for you, you like…will exercise makes you happy or not? Do you like exercise?

Amirah: Not very lah. Sometimes when we exercise in the afternoon, by the time we get back to our room we are tired. So, at night cannot do that much work. Sometimes I even fall asleep.

Hanisah: Okay, what kind of exercise which you like?

Amirah: Zumba. Aerobic which is light. No need to go jogging, running. Ha.

Hanisah: If like stretching, ah, up and down stairs, rope jumping?

Amirah: Ah. Can. Like.

Hanisah: Um, we wanted to produce one exercise module for females who stays in KTSN. You want to join or not? And then what makes you want to join?

Amirah: If that thing is interesting and attracting, ah, will join.

Hanisah: What do you want to be included in the exercise module?

Amirah: In the exercise module…like just now Zumba, aerobic, stretching that kind.

Hanisah: Um, if we provide guideline in the exercise module and ask you to exercise alone for a month. So in that time, you will be in control, not necessary taking up a lot of time. Are you willing?

Amirah: Um, feeling not because if there is people to guide its more easier to do. If alone like hard abit to do.

Hanisah: If we ask you to exercise every 2 days, meaning that you exercise then you rest, then you exercise, then you rest then you exercise back. Are you willing?

Amirah: Can.

Hanisah: Okay, uh, what can motivate you to exercise that, like frequently.

Amirah: Motivation eh?

Hanisah: yes.

Amirah: If that module like in the end got effects on ourselves meaning reduce weight or others.

Hanisah: Okay, uh, should we include running in the exercise module?

Amirah: No. Hahahaha, no.

Hanisah: Okay. Last question. What is your goal when exercising? You want to get fit, build muscle or lose weight? Or there is other goal?

Amirah: If exercise normally lose weight and build muscle.

Hanisah: Okay, thank you for joining.

Transcript Respondent 20
Name: Nurul
Age: 23 years
Faculty/Program/Year: FSK/Biomed/4
Respondent Code: R20

I: Assalamualaikum Warrahmatullahiwabarakatu.

S: Waalaikummussalam.

I: I am Ida, I will ask about few questions regarding exercise activities. This session will be recorded. Will you give the permission to do so?

S: Yes. Can. Can.

I: Question. You are Nisha right? Is Nisha doing exercise routinely?

S: So far no.

I: No. Okay. Nevermind. What does Nisha understands about physical avitivity?

S: Physical activity, activity which involves our physical body.

I: What does Nisha understand about exercise?

S: Exercise. Exercise. In my opinion, whatever activity which involve body movement. It is exercise for me.

I: How Nisha distinguishes physical activity and exercise?

S: Aaa. They are not the same thing?

I: Nisha feels that it is the same thing.

S: The same thing.

I: Okay. It’s okay. Why Nisha does not carry out exercise?

S: Why don’t carry out?

I: Haah.

S: First maybe lazy. Then feel like time restricted.

I: Time restriction is caused by?

S: Study, assignments, FYP, haah about this.

I: What else?

S: What else?

I: Is there any more reasons?

S: We feel that ourselves are healthy.

I: Is not because of no friends?

S: No.

I: Or no place to execise?

S: Aaa. Actually there is a lot of place to exercise. Haah. We can exercise also in our rooms.

I: What does Nisha do not like about exercise?

S: Make us tired, sweating. Aaa that’s why.

I: Only tired and sweating. Don’t mind about the pain?

I: That’s it. in Nisha’s opinion, what is the exercise benefits for us?

S: What benefits?

I: Haah.

S: Exercise makes our body healthy right?

I: Yes.

S: Invigorates the mind, invigorates the body. Besides that, our metabolism can function properly.

I: How about stress releasing through exercise?

S: Agreed also. Exercise can release stress.

I: Okay. That’s it?

S: Haah.

I: If Nisha wants to start exercise, what makes Nisha to start exercising?

S: Meaning what can trigger us to exercise?

I: Ya.

S: Maybe from peers, they invite us to exercise “Wei, lets exercise.” Secondly, when we feel that our body is weak, or feeling sick just will start to exercise.

I: How about exercise motives? Like some people exercise to maintain their appearance, lose weight like that?

S: For Nisha, we exercise to take care of the body, it's a cheating to say not to. To take care of our body so that our body is normal.

I: Do Nisha felt before that exercise is exciting and wanted to repeat it? Will Nisha starts exercising because it is interesting?

S: Ya.

I: When Nisha start to exercise, what will cause Nisha to follow the exercise willingly and continue to do so? For example, when some people started the exercise already, they will stop after a few days. How to continue to exercise?

S: We need to have consistent schedule. For example, in one week, three times for about 10 minutes. We need to have a schedule.

I: Anymore? Like friends or environment?

S: Environment depends on the weather, if raining, can do exercise in indoor right. The important thing is schedule, like one week 3 times. Consistent.

I: Okay. How about friends. Do you feel that friends can help in maintaining exercising?

S: Yes. They can become our exercise companion so that we are more motivated because of friends.

I: How about self-awareness? Like some people they keep on exercise because they are not healthy, they have the self-awareness to become healthy.

S: Meaning that self-awareness is important. We need to understand that we are not always healthy so we need to exercise. Yes, that is self-awareness.

I: Which type of exercise Nisha likes?

S: Cycling.

I: Cycling?

S: Yes, as long as got movements.

I: What if jogging?

S: Jogging, not so.

I: Not so. Up and down stairs?

S: Can.

I: Can. Sit up?

S: Can.

I: If Zumba, yoga?

S: No.

I: If sit up?

S: Interested.

I: Push up?

S: Can also.

I: Can also. If stretching?

S: Stretching. Isn’t stretching important? Ha.

I: Okay. That’s it?

S: Haah.

I: Okay. If we wanted to have an exercise program, what makes Nisha wants to follow the exercise program? What characteristics of the exercise program can make Nisha interested to join?

S: First, it needs to be interesting. With a schedule, meaning that not only focusing on exercise, with talks also, with lucky draw, provides gifts for everyone and then separate into male and female for exercise. Not mixed.

I: If we come out with a exercise book, in the book there is daily guideline on how to exercise?

S: Haah. Good.

I: Good or not? What are the characteristics of exercise which Nisha would like to do?

S: Characteristics of exercise. Meaning?

I: Meaning is that exercise can be done in the room or not like that.

S: The characteristics must be easy to be done.

I: Easy to be done?

S: Meaning that it can be done in room, indoor or outdoor.

I: How about the exercise must be easy to follow?

S: Haah. Easy to follow. No difficult twisting moves like Zumba.

I: That is it?

S: Haah, that is it.

I: If the exercise module needs Nisha to exercise today and rest tomorrow and exercise again the next day. Is Nisha willing to follow?

S: If it is necessary for us to do so, Nisha will follow.

I: Nisha feel that in a day, how long is Nisha willing to exercise?

S: Maximum 30

I: minit?

S: Haah. 30 minit.

I: Okay. What makes NIsha willing to maintain exercise in interval days?

S: Meaning our motives?

I: like our motivation or any goals?

S: That is it. With a goal. Our goal like today do, tomorrow stop, next day resumes. Meaning that we haven’t got what we wanted. For example, like wanting an ideal body, weight below 50, 45. These type of things makes us to do continuously. Consistent right?

I: If we already reach the objective, how can we maintain?

S: Wanted to maintain after reaching objective. We ah! Maybe after a lot of times exercising, we are used to it so even if we reach the target or objective, we will still continue due to getting used to it. Some people are like that.

I: If like the thing is exercise or the results can be seen, will it makes Nisha continue to do so?

S: Haah. Right. When we see the results, it will be fun to continue the exercise.

I: Okay. That is all the questions. Thanks.

Transcript Respondent 21
Name: Syazani
Age: 22 years
Faculty/Program/Year: FSK/EVH/4
Respondent Code: R21

Hanisah : Can we record?

Syazani : Okay

Um, okay. I'm Hanisah who will be the interviewer and eho's the interviewee?

Syazani : Uh, I am Syazani

Hanisah : Okay, um, define physical activity for you

Syazani : Physical activity… rugged activities, maybe the ones that we do outdoors. More outdoorsy, unlike pilates, youg, those are in a room. If physical activities, maybe it;'s more ourdoorsy.

Hanisah: Okay, uh, define exercise

Syazani: Exercise is the movement that we commonly do. Like the ones at home, we walk to the front, to the back, clean the house, lift heavy items, well not that heavy, light ones, and also the common ones, maybe youga would be considered as exercise.

Hanisah: Okay. Okay. How is physical activity different from exercise to you?

Syazani: It's kind of the sama, but I Think exercise, it's more relaxed, doesn't use too much energy. Physical activity, it's like activity.. what? Use more energy like when we go hiking. Ha, that uses a lot more.

Hanisah: Okay, ah dou you exercise routinely? 2-3 times per week

Syazani: To tell you the truth, I do not exercise regularly. Hahaha more like never.. ha..hahaha

Hanisah: Okay, it's alright. Ah, what are the reasons why you do not exercise?

Syazani: I'm lazy, I'd rather waste time watching stories on my laptop than exercise. Because when you exercise, you sweat, then you have to change your clothes and shower…its extra effort, I'm lazy and I don't like it. Hahaha.

Hanisah: Okay, um, why else you do not like to exercise?

Syazani: Why else I don't like? Well it's not that I don't like. Because of the sweat. Sweating would take time, sometimes tiring, Very tiring.

Hanisah: Um, do you think exercise is good for you and how it is good for you?

Syazani: Come to think about it, exercise if the best especially if we do it regularly. Routinely or not, at lease we do it but um, I don’t do it. I think it’s good. Haha

Hanisah: Okay, um, what can we do to ensure you start exercising?

Syazani: What can we do? You organize, um, start aerobics..

Hanisah: Apart from specific programs, such as aerobics or Zumba, is there anything else we can do to increase your motivation to exercise?

Syazani: If it was like before this, when I had mood to exercise, maybe I would buy a skipping rope, put it in my room so that I can use it every day. That’s all that I can think of that can increase my mood to exercise. We can buy exercising equipment to help us exercise.

Hanisah: Ah, okay. Um, what kind of exercise do you like?

Syazani: I like skipping, um, on-the-spot jogging, walkinh, walking in… hmmmm *clears throat* That’s it.

Hanisah: How about Zumba? Aerobics? Stretchings?

Syaza ni, Stretching is okay, Zumba aerobics are okay too in the long run. Uh, it has to be short enough. Hahaha. Because if it’s too long, we cannot follow the actual movements, it’s hard. And one more thins, make sure the steps are not difficult. If it’s difficult, it’ll be difficult to follow what was taught by who?

Hanisah: Instructor?

Syaza ni: yes, what instryuctor do.

Hanisah: Haa. Okay, um, okay. We are planning to produce an exercise module for KTSM ladies, uh, do you want to join? And then, what would like to be included in the module?

Syazani: Um, exercise module? I am okay with joining the module, but it will depend on whether the time is compatible with me, right? Sometimes, when busy, there will be no time to join. What could be included? Um, the usual stretching, then with the aerobics, the steps should be pre-determined steps. Zumba is also okay. Um, jogging is okay too. Mass jogging, it won’t be too boring. That’s all.

Hanisah: Okay, um, for the exercise module if we provide you the guidelines and then we give it to you to do on your own. Mostly you will be exercising on your own, in your room, anywhere you are comfortable, and then on certain times, we will call you to exercise together. Are you still interested?

Syazani: Um, if guideline is provided for us to do it on our own, that will a bit difficult. Because even with the equipernts that we buy ourselves, we still just keep it without using it. Even we buy using our own money. If the guideline is provided, I probably won’t do it on my own. Hahahah. That’s it.

Hanisah: Okay, um, if we ask you to exercise every two days? Like exercise, not “exercise” exercise. So is that too frequent for you?

Syazani: Uh, for me that is too frequent. Maybe after lapses of 2 days? So we will have a long duration of rest, well, not too long, at least , ah 2-day rest, then continue, 2-day rest, then continue. I think that would be better.

Hanisah: Okay. Um, cam we include running in the module exercise?

Syazani: Running? Do you mean long distance running?

Hanisah: Um ..anything. It could be a 5 minutes run, or it could be a 15 minute run. What do you think? Which one do you prefer?

Syazani: Haha, if running, I usually run around the fiels, and one round is alreade making me breathless, like that. If I run for 5 minutes, it's unlikely hahahahah. At least if it's jogging, if jogging, it will be ok.

Hanisah: Um, ok. Um, last question. What is your goal when exercising? Do you want to get fit? Lose weight? Build muscle? What?

Syazani: Not so much for weight loss. More like, um, increase what? Cardiovascular in the body. Our heart beat.. better.

Hanisah: So it's to get fit?

Syazani: Ha, get fit. I think that's it.

Hanisah: Okay. Okay, thank you for joining the interview.

Transcript Respondent 22
Name: Murnie
Age: 23 years
Faculty/Program/Year: FSK/Biomed/4
Respondent Code: R22

Hanisah: Okay. This is Hanisah, ah, can I record the interview?

Murnie: Yes

Hanisah: Can you please give me your name?

Murnie: My name is Murnie

Hanisah: Okay Murnie. What is physical activity?

Murnie: In my opinion, physical activity is any movement that involves movement of feet and hand, regardless any activities

Hanisah: Okay, what is exercise

Murnie: I think exercise is lia a specific movement, like jogging, cycling, or with movements like sit-ups, ah, easy said, it's any specific movements

Hanisah: What is the difference between physical activity and exercise

Murnie: I think physical activities are lighter than exercise. That's it.

Hanisah: Okay, ah, do you exercise routinely, I mean 2/3 times a week, every week?

Murnie: No. I used to, now not anymore. Used to

Hanisah: Okay. Uh, any reasons why you do not exercise routinely

Murnie: Ah, one reason is because I do not have self motiovation. Ah, secondly, lazimess. Thirdly, always busy. Fourthly ah because I do not have a partner to exercise. Fiftly, I like to sit as opposed to walk and exercise. Ah, that's it.

Hanisah: Ah ok. What are the things you do not like about exercising?

Murnie: Ah, I actually like exercising. Uh, but I don't like it when people watch me exercise. I seriously don't like it, not at all. Sweating is fine and all it's just that I don't like it when people watch me exercise.

Hanisah: You have that feeling, ah, ashamed of your body appearance?

Murnie: Ah, at the moment no such feeling. Hahaha. So far it's okay. Not that content, but not ashamed. Just okay.

Hanisah: Why don't you like people watching you exercise?

Murnie: I feel inferior, introvert and not confident with myself probably. Ah, maybe, ah.

Hanisah: Okay. How do you think exercise is good for yourself?

Murnie: Um, I think exercise can help make our body fitter, to maintain BMI, body shape, then we can control our blood sugar level, then we can remove the toxins and we can be happy when we exercise.

Hanisah: so you are happy when you exercise?

Murnie: Very. Happy. Very much.

Hanisah: Okay, um what can be done, ah, to make you start exercising.

Murnie: Um, maybe we can start setting a specific schedule and monitoring. Ha, then we need constant encouragement or we have a partner to exercise together. Maybe we can be exercise constantly. Ah like that.

Hanisah: Okay, um, do you prefer to exercise on your own in your room like, in your own comfort zone? Or mass exercise in an activity with a set timing?

Murnie: Um, I prefer the second option. Hahaha

Hanisah: Why?

Murnie: Because there are many people in a group. The more people there are, the more motivated I feel. When we see other people motivated, we also feel like, oh, we want to do that too but when we are alone, we feel like lazy lah, do tomorrow lah, tomorrow. We procrastinate it until we eventually end up not doing it. Hahahaha

Hanisah: Ah. Okay, um, what kind of exercise do you like?

Murnie: Ah, I prefer…um.. Zumba I don't quite like. More towards cycling, I like cycling. Ah, I like swimming too, and then, ah, I don't quite prefer running. Tiresome. Hahaha. But jogging is okay, more, I don't know what else I like. But I like static exercises, ah, like that. Static.

Hanisah: Okay, um, are going up and down the stairs, or stretching, stuff like spot jumping, okay?

Murnie: Ah, I have a skipping rope in my rome, so I frequently skip in room. But it's just once in a while, almost everyday but not much repetition, sometimes I do 10, sometimes 20 a day. Ah, so that's it.

Hanisah: Okay, that's good. Ah, later, we will produce an exercise module for ladies living in KTSN, would you like to join? And if you join, what would you want in the module?

Murnie: InsyaAllah I would like to join, because if you are producing it for us, we feel more welcomed, right? Um, what I want? Um, ah maybe apart from daily supervision to check if a person exercises, maybe you can make a schedule. Today, what do you have to do, how many sit-ups do you have to do. The thing has to be organized. How many times do you have to do something, how many minutes do you have to do it, how do you do it, like that. Like, I actually want to exercise but I don't know how, like for this, should I do this thing first, or that thing first. Should I do sit ups first, or this other thing

first. I don’t know the basics of exercise. I do watch videos on YouTube® and others but sometimes I do them and other times I don’t. If we can produce something that’s like organized and okay, Insya Allah I can follow it.

Hanisah: Ah, okay. Um., would you exercise every 2 days? Meaning, you exercise, then you rest, then you exercise, then you rest, then you exercise. Is that too frequent?

Murnie: Um, to me that isn’t too frequent. It depends on how busy I am as well. If I’m not busy, exercising every 2 days is fine.

Hanisah: Ah, what will make you more motivated to exercise every 2 days?

Murnie: Uh, when I have a partner. Hahahaha

Hanisah: Okay, uh, should we include running in the exercise module?

Murnie: Yes. Should, should.

Hanisah: But you said you don’t like running.

Murnie: Ah, it’s not a problem. But I don’t quite prefer. But if it’s included, I can just do it. Hahaha

Hanisah: Ah, what do you thino of the running limit, like running too long, is 5 minutes too long to you? What limit do you want to impose?

Murnie: Above 15 minutes is too long. Ah, 0-15 minutes is okay.

Hanisah: Okay, last question, ah, what is your goal when exercising? You want to become thinner, build muscle, get fit? Which one?

Murnie:, Ah, probably to get fit. Building mucle, probably not. Getting thinner is probably one of the aim, but to become healthy is another aim. First aim. For health.

Hanisah: Ah, okay, thank you Murnie.

Transcript Respondent 23
Name: NurMaisara
Age: 23 years
Faculty/Program/Year: FSK/Opto/4
Respondent Code: R23

I: Assalamualaikum. I’m Ida. I will be asking a few questions about exercise. This session will be recorded. Do you agree to join this session?

S: yes

I: Maisara, right? Can I call you Mai? You do it routinely?

S: no.

I: What do you understand about physical activities?

S: Physical activities, self-exercise, maybe.

I: Exercise

S: Yes. Exercise.

I: Exercise. What do you understand about exercise?

S: Exercise involved body movement, physical movement, in which, maybe able to draw sweat , like that

I: How do you distinguish between exercise and physical activities?

S: Exercises causes us to sweat, physical activities is just like walking, like body movement.

I: Why don’t you do physical activities?

S: Activity and exercise are the same right?

I: The same. The same

S: Maybe time constraint. Time constraint, maybe and maybe there’s no interest.

I : No interest

S: Yes, no interest

I: Like..

S: No desire to do that

I: Is it because of place or because there’s no equipment?

S: Not really. For me, if there’s interest.. and. Interest. If one is interested, she will join, for example, when hungry she will be interested. Maybe I’m not interested.

I: What don’t you like about exercise?

S: What I don’t like about exercise. It’s tiring maybe.

I: Tiring. What about sweating, aches?

S: Haa. That’s it. Aches.

I: What do you think the benefit of exercise to you?

S: Benefits of exercise, probably because it could. Burn calories, healthy, health stability. Yes for our health.

I: Health for yourself.

S: Yes

I: If you want to start exercising, what would be the trigger to do it?

S: Perhaps, invitation from friends is important.

I: Invitation from friends

S: Maybe, because.. Maybe I’m in a circle of friends who do not exercise. So they never invite. Then I’ll have no partner to exercise. No friend.

S: Yes. No friend. What triggers me, if I’m invited to play netball, I will play. But if nobody ask, I will not play.

I: How about your objective

S: Objective?

I: Yes

S: Objectives of exercise?

I: Yes

S: Objectives of exercise

I: Do you have any objectives that trigger you to exercise?

S: Ohh! Maybe. Reason?

I: Yes

S: Reason that trigger awareness

I: Self awareness

S: Yes

I: You want to be healthy

S: Or, you can suggest an answer? So I don't have to think much

I: Usually, other would answer the objective to be healthy or to lose weight, self appearance.

S: Maybe for health. If it's for me, probably health is the reason for me to start exercising. I will exercise.

I: Once we have started exercising, sometimes,after starting exercising. Then after a few days she become lazy to continue. What would make you to continue exercising?

S: What would make me continue? The healthy feeling itself.

I: Healthy feeling itself

S: Yes

I: Is feeling healty something like more energetic?

S: Yes. Energetic body will lead to better focus during studying. Actually, you can focus during exercise. Probably, it creates the effect from the exercise activities itself.

I: is that all?

S: Yes

I: In your opinion, what is your favourite exercise?

S: Sports maybe. I mean games. Not just jogging. Maybe netball, badminton. Exercise like thatm not just jogging. Not weight training.

I: No weight training. If..

I: Jumping, rope skipping?

S: Haa, those are okay

I: How about going up and down the stairs

S: Hmmm. That's why I said I prefer exercise that involves games. Because, if it's like that, it's individual right. It's not fun.

I: In your opinion, do you prefer exercise in group or alone?

S: Haa yes

I: In group

S: Yes with friends. If I do it alone, it'll be difficult. But if someone ask me to jog together, I'm okay with it. No friend, I will not do it.

I: If we were to develop an exercise, what would make you be interested to join?

S: Feeling healthy. If I feel the exercise help make me energetic, less sleepy, something like that, and the activity should be fun.

I: Activities that are easy to follow or can be done with friends without help.

S: Yes, easy to follow. Correct

I: If the exercise module requires you to exercise, exercise today, then rest on the next day, and then exercise again. Would you be willing to do it?

S: The module?

I: Yes

S: We'll see, if I have time

I: See if time permits. Usually if you exercise, in a day, how many minutes do you allocate for it?

S: Because I rarely exercise, if I exercise, it's less than 1 hour.

I: What would make you continue exercising every other days?

S: What would make me continue exercising every other days?

I: Yes. Maybe, the exercise module itself, of friend factor or condition of the exercise itself.

S: The condition of the exercise itself, if I feel comfortable, if I can join the exercise and if I have friends along. If I'm doing it alone, it'll be difficult.

I: That's it.

S: Yes

I: Thank you

Transcript Respondent 24
Name: Izzati
Age: 23 years
Faculty/Program/Year: FSK/Biomed/4
Respondent Code: R24

I: Assalamualaikum, I'm Ida

S: Waalaikumsalam

I: This is Izzati right?

S: Yes, Izzati

I: I am going to ask a few questions about exercise activities. This session will be recorded. Do you agree?

S: I agree

I: The question is, have you done exercise activities leisurely or regularly?

S: I have done exercise before but not frequent, about once a month. I usually jog.

I: So it's not routinely done

S: Yes, not routinely

I: What do you understand about physical activities?

S: What do I understand about physical activities, what we do to fit our body, it doesn't matter if it's light or heavy activities. For example, skipping, jogging, swimming, like those involving movement.

I: What do you understand about exercise activities?

S: Exercise. Jumping, and movements.

I: Movements

S: Yes movements

I: How do you differentiate between physical activity and exercise activity?

S: I don't know

I: You don't know. It doesn't matter. Okay. What are the reasons you do not exercise?

S: I don't have time, actually. Pressed for time.

I: Pressed for time, what causes it?

S: Studying, doing my final year project.

I: Any other reasons? Because your friends do not invite, or you do not have place?

S: They have. If alone, I will not go. I will go if I have friends with me.

I: If, you are not motivated to exercise?

S: No

I: No

S: No

I: What don’t you like about exercise?

S: I like exercising just I don’t practice it often. I like the sweaty feeling, feels healthy. I like

I: Sweat, healthy you like. Nothing that you don’t like. Okay. In your opinion, what are the benefits of exercise to you?

S: Firstly, it cam make me slimmer. Then we feel healthy, make use feel more energetic, feels like I have high stamina

I: High stamina. Okay sure, sure. Is that all?

S: Yes. That’s it

I: That’s it. How about you want a healthier body and have time to do it

S: Have time, probably not. Healthy body, one of the reasons.

I: Okay. What can trigger you to start exercising?

S: Increased body weight. Then I feel like exercising. One more thing, if I feel stressed and need to release my tension I usually go jogging and it feels like tension is released

Okay. What if, your friends or if there’s free exercise session at your college?

S: Do I go?

I: Yes, Do you go. Would it trigger you to exercise?

S: Not so much. Because I not a person who only join when there’s an event. If I want, I’ll go on my own. I don’t like events.

I: What could make, like goal, or motivation, you stay exercising?

S: The fun, like the satisfaction when we exercise that makes us want to exercise more and more.

I: Anything else? Maybe the benefits of the exercise or gaining new friends or to achieve our goals?

S: I agree with the reason to get healthy body because after exercise, we will feel healthy, sweaty. Also, if we go with friends, we can improve friendship.

I: What kind of exercise you like to do?

S: I usually jog or cycle.

I: Cycle. How about rope skipping?

S: I rarely do that because I do not have skipping rope.

I: How about going up and down the stairs

S: Only when needed. When there's no lift

I: So you do not like it

S: I don't like

I: How about push-ups?

S: I don't do push-ups

I: You don't like sit-ups also?

S: I don't do

I: Stretching?

S: No

I: No. okay

S: Because I don't exercise much.

I: It's okay, it's okay. If you want to exercise, do you like exercising alone or in the room or in groups?

S: In group, but not a big group. Like two or three persons, if with a lot of people, I don't like

I: If we were to develop an exercise, wait, no no. I Zumba, do you like?

S: Just okay

I: Just okay. How about yoga?

S: Yoga sounds interesting but I've never done it. But interesting.

I: If we were to develop an exercise, what do you think that would make the exercise appears attractive to you to join?

S: I don't know.

I: Is it because the program is easier to follow, or it's interesting or if many of your friends are joining?

S: That's true. If the program is attractive according to my interest, and there are friends joining along maybe I will join.

I: If the program is the one that we provide a guideline, you and your friends can follow the guideline, and the guideline is easy to follow, and you can do it according to your own time. D you want to join?

S: InsyaAllah, if I have free time,I will join

I: Usually, when you exercise, how much time do you spend to exercise?

S: Around ½ hour to 45 minutes. Not even 1 hour

I: The module we're developing, if we require you to exercise today, rest tomorrow, exercise again the next day. Are you willing to do?

S: Ahh. If I have time.

I: If you have time?

S: Ha. If I have time

I: I you have time. What would make you continue exercising every other day? Sometime, some people would exercise one day, then rest and then continue resting. How do you prevent such things?

S: To prevent from suddenly quitting?

I: Yes. Like how to push ourselves

S: Need motivation

I: Motivation

S: Without motivation, tomowwor we do and then we stop.

I: Is there anything else?

S: No

I: Like friends, or you need a guide.

S. A guide would be good. Friends may be useful for motivation

I: Okay. That is all the questions. Thank you

S: You're welcome.

Transcript Respondent 25
Name: Rosmawati
Age: 22 years
Faculty/Program/Year: FSK/Nutri/4
Respondent Code: R25

Hanisah: If I record this interview, is it okay?

Ros: Sure, sure

Hanisah: Okay, I am Hanisah. Uh, can you state your name?

Ros: Rosmawati binti Dora. Haha

Hanisah: Define physical activity

Ros: Do I have to speak in English? Hahaha

Hanisah: Haha, malay Language is fine

Ros: Physical activity.. exercise. Something physical? Define? When you move, it's physical activity. Haha, logic

Hanisah: Ah, what is exercise?

Ros: Exercise is.. when.. what? Something organized. For example aerobics. Something like that

Hanisah: Okay. Is physical activity and exercise the same? Or are there any differences?

Ros: Physical activity is general. Such as sitting, standing is considered physical activities but it's not exercise.

Hanisah: Perfect answer.

Ros: Hahahaha. I learned it.

Hanisah: Do you think you exercise routinely? 2-3 times a week, at least 30 minutes at a time?

Ros: Ah, no

Hanisah: Okay.ah, what are th reasons you do not exercise?

Ros: Because, firstly I do not have the time. Secondly, I'm lazy. Hahaha. Ah, those are the reasons

Hanisah: Okay, it's fine. What don't you like about exercise?

Ros: Ah, firstly because it's tiring. Secondly, ah that's it. Because after exercising.. I usually exercise st most for 15 minutes. After that 15 minutes, I became tired, ah, and go straight to sleep

Hanisah: Okay. Um, what can you do to make you start exercising? To motivate you

Ros: To lose weight. Hahaha. That’s the main reason

Hanisah: Um, what can other people do to motivate you to exercise

Ros: What other people can do? Oh, is someone invite me to join. Like going to the lake, jogging, ah, that sometimes can motivate me. Or when people said that I’m fatter. Hahaha. That will motivate me. Hahaha

Hanisah: Okay, what kind of exercise do you like?

Ros: aerobic exercise. Zumba. Haha

Hanisah: Um, what else do you like? What if jogging, running, going up and down the stairs? Stretching?

Ros: Probably not running. Moderate jogging is ok. Walking. Ah, walking. Hahaha, that is okay. Climbing up and the stairs, noo. Hahaha

Hanisah: Okay, um, how about rope jumping?

Ros: Rope jumping, maybe not. It is easily tiring.

Hanisah: Ah, ok. Um, we are producing an exercise module next semester for KTSN ladies. Ah, do you want to join the program? And if you do? What do you want to be included in the exercise module? Just imagine the module is like we give you the guideline and then you do it on your own in your own room and then it’s up to you, at what level. And also, some of the activities are in groups.

Ros: Wait, does it mean that it’s a daily module that we need to do? Like that?

Hanisah: Yes, 2 times in a week

Hanisha: Half an hour

Ros: Based on activity that.. meaning? The exercise that we do based on what is given? We cannot do our own exercise?

Hanisah: Yes

Ros: Oh… then what else you want? Is it example?

Hanisah: um, do you want to join? And what do you want to included in the exercise module?

Ros: I am quite interested. What else you want? Hahahaha,meaning? You want activities right?

Hanisah: Ah, whaat activities that you think are best included in the exercise module

Ros: Zumba. Hahaha. And what else? Now there’s that exercise? The one that.. sometimes there’s a 5 minute plank, something like that. I don’t have the name

Hanisah: Like the static exercise?

Ros: yes. The sit-ups, ok. Sit ups for 10 minutes, something like that

Hanisah: Ah, I understand, I understand

Ros: Ha, something like that

Hanisah: Okay, um, if we ask you to exercise every 2 days, meaning you okay, um, if we ask you to exercise every 2 days, meaning you exercise, then you rest, then you exercise, then you rest, then you exercise, is that too frequent?

Ros: No, it's okay. Not that frequent

Hanisah: Um, what makes you feel that it's okay, not too frequent? What makes you motivated to exercise that much?

Ros: Because this is for 5 days, right? Because it's 2 times a week. 2 times a week is considered infrequent. It should be 3 time a week. So it's ok, it's considered minimum. Minimum, hahaha

Hanisah: Okay, um, should we include running in the exercise module?

Ros: Running? I don't think so. Hahaha. Rope jumping is probably okay. Sometimes some people do not want to go out from their room. Haha, you can't expect them to run in the room. Hahahaha

Hanisah: ok, I understand. Okay last question. Ah, when you exercise, what is your goal? To get fot, build muscle, shape your body, ah, lose weight, um, is that it?

Ros: Actually, the main reason that I want to exercise is to reduce my body weight because of my fat. I have a high fat concentration, so I want to shape some muscles lah. Because every time I check, it's always higher than it should be. So that's the real reason that I want to lose body weight.

Hanisah: Okay, thank you for joining.

Transcript Respondent 26
Name: Wendy
Age: 23 years
Faculty/Program/Year: FSK/Nutri/4
Respondent Code: R26

I: Hi Anne. Is that Anne, Wendy. I will be asking a few questions about exercise activities. The session will be recorded. Is that okay?

S: Yes

I: Wendy. Do you do routine exercise activities?

S: Sometimes but not routinely

I: Sometimes but not routinely. What do you understand about physical activities?

S: Physical activities are movements the we do with our body. That’s physical activities for me

I: How about your understanding about exercise.

S: Exercise is part of physical activities except that exercise is more organized. Organized such as aerobics ?

I: How do you differentiate between physical activities with exercise?

Physical activities, for example can exercise, but exercise sometimes cannot be considered as physical activity because physical activities are movement, movements that we make. We lift things or opthers are physical activities. For exercise, it would be more organized.

I: Organized. What are the reason you seldom do exercise routinely?

S: Because I do not have time

I: No time

S: Yes. That’s it and laziness

I: No time, why no time?

S: No time because I’m a student. Always busy, what with being in the final year.

I: Can it also because no invitation from friends or if the facilities in the college is not satisfactory that causes you not to exercise?

S: Actually, it’s all there. But the realy thing is that I lack self-motivation

I: Lack of self motivation. What don’t you like about exercise?

S: It’s tiring. And after that, after a day, for example, if there is motivation to exercise today, tomorrow I will lose motivation. That’s why I’m tired.

I: Tired. Okay. Can it also because of sweating or aches after exercise?

S: No

I: No. okay. What do you think is the benefit of exercise to you, yourself?

S: Actually, exercise, even though I said it tiring, but the effect is that it gives more energy

I: Apart from giving energy?

S: Apart from giving energy, it make us feel fresher. Fresh and energetic

I: That’s all

S: That’s all

I: If you were to start exercising, what could be the trigger for you to start exercising?

S: The trigger, firstly self motivation, secondly, friends

I: What about the first

S: If the first one is not there, no self motivation, even if friends invite to join, I will not join

I: Self motivation is important.

S: Most important. Most important

I: Friends come second. Does it mean goal is not that important?

S: Goal, not so much

I: When you start exercising, what really help you to maintain to continue exercising?

S: Continue doing exercise. What..

I: Helps you to maintain routine exercise

S: Self motivation

I: How about, if there are friends who invite to join, or any goals?

S: I do not set any goal. What’s important is that I exercise and sweat, all that.

I: It’s for exercise, for self motivation

S: Yes. Correct

I: What kind of exercise do you prefer?

S: Exercise. I am not the person who likes to go to the gym. I like badminton or sports, like that

I: sports like that. How about jogging?

S: Jogging. I seldom jog. Don’t really like it.

I: don’t really like it. How about cycling?

S: Cycline is good too, but depends on the facilities

I: Okay. Zumba?

S: Zumba. Not so much

I: Not so much. Yoga, not so much also?

S: I’ve never tried yoga, but I kinda don’t like it.

I: How about climbing yup and down stairs? You like?

S: Climbing up and down the stairs. I’m okay with it. Climbing up and down the stairs are still okay.

I: Still okay. Sit ups?

S: Sit up. That, I don’t quite like

I: Don’t quite like, Push ups you also do not like?

S: No

I: If we were to produce an exercise module, what would attract you to join the exercise module?

S: It’s really up to the exercise itself

I: The exercise, what kind of exercise that you want?

S: What kind of exercise. Okay. Even though I said I do not like activies such Zumba, aerobics, but if those are included, it’s probably okay. Maybe I will join

I: The exercise will be a mass exercise, would you join?

S: Haa. That I will join. Because if we exercise in groups, that is better that exercising alone

I: If we give you a guideline fou you to exercise, according to your own time, in your own room. Would you want to do it?

S: Yes. Because if everything is provides like what you said just now, then there’s no reason to say that there’s not time to do.

I: Do you prefer exercising alone in the room or in group?

S: 50-50

I: So it’s neutral

S: Neutral

I: You think how long are you willing to spend time exercising in a day?

S: 30 to 1 hour

I: 30 to 1 hour. If we develop the module that requires you to exercise today, rest tomorrow and then exercise again. Would you be willing to do it?

S: I’m willing because I don’t like doing it everyday. Have to do everyday

I: Not every day

S: Not my preference. If I do it today, then rest tomorrow. After that, I’ll do it again.

I: Why do you prefer it like that

S: Because I want to rest

I: You want to rest. What would make you want to keep exercising eventhough there are rests, rests rests?

S: Myself and friends

I: Yourself and friends

S: Maybe also include the goal to become more fit

I: Goal to be more fit, no reason to lose weight or to maintain self appearance?

S: No

I: No. Thank you. That’s it
